# Identification of a Novel Structural Class of H_V_1 Inhibitors by Structure-Based Virtual Screening

**DOI:** 10.1021/acs.jcim.4c00240

**Published:** 2024-06-08

**Authors:** Martina Piga, Zoltan Varga, Adam Feher, Ferenc Papp, Eva Korpos, Kavya C. Bangera, Rok Frlan, Janez Ilaš, Jaka Dernovšek, Tihomir Tomašič, Nace Zidar

**Affiliations:** † Faculty of Pharmacy, University of Ljubljana, Aškerčeva cesta 7, Ljubljana 1000, Slovenia; ‡ Faculty of Medicine, University of Debrecen, Egyetem tér 1, Debrecen H-4032, Hungary; § HUN-REN−UD Cell Biology and Signaling Research Group, Egyetem tér 1, Debrecen H-4032, Hungary

## Abstract

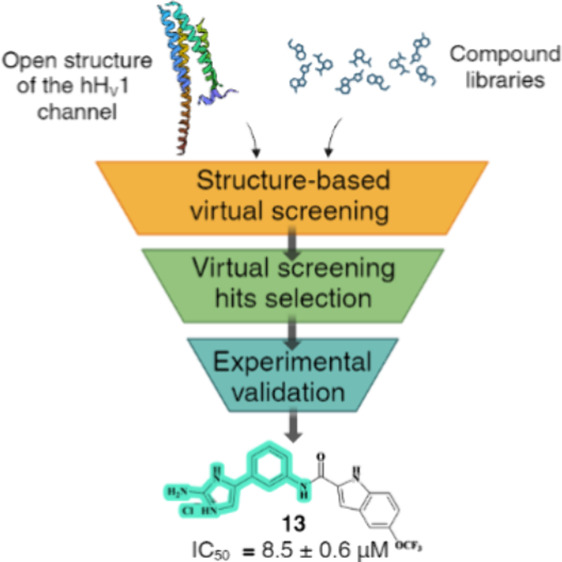

The human voltage-gated proton channel, hH_V_1, is highly expressed in various cell types including macrophages, B lymphocytes, microglia, sperm cells and also in various cancer cells. Overexpression of H_V_1 has been shown to promote tumor formation by highly metastatic cancer cells, and has been associated with neuroinflammatory diseases, immune response disorders and infertility, suggesting a potential use of hH_V_1 inhibitors in numerous therapeutic areas. To identify compounds targeting this channel, we performed a structure-based virtual screening on an open structure of the human H_V_1 channel. Twenty selected virtual screening hits were tested on Chinese hamster ovary (CHO) cells transiently expressing hH_V_1, with compound **13** showing strong block of the proton current with an IC_50_ value of 8.5 μM. Biological evaluation of twenty-three additional analogs of **13** led to the discovery of six other compounds that blocked the proton current by more than 50% at 50 μM concentration. This allowed for an investigation of structure–activity relationships. The antiproliferative activity of the selected promising hH_V_1 inhibitors was investigated in the cell lines MDA-MB-231 and THP-1, where compound **13** inhibited growth with an IC_50_ value of 9.0 and 8.1 μM, respectively. The identification of a new structural class of H_V_1 inhibitors contributes to our understanding of the structural requirements for inhibition of this ion channel and opens up the possibility of investigating the role of H_V_1 inhibitors in various pathological conditions and in cancer therapy.

## Introduction

Voltage-gated proton channel 1 (H_V_1) is a transmembrane protein that was first described more than 30 years ago, however, the channel gene (*hvcn1,* hydrogen voltage-gated channel 1) was not identified until 2006.^1−4^ H_V_1 is expressed in various immune cells, skeletal muscle cells, oocytes, osteoclasts, blood cells, sperm cells and DRG neurons.^1,5−13^ H_V_1 channels are exclusively selective for protons, conducted through the voltage sensing domain (VSD), in which aspartate and arginine residues are responsible for the high proton selectivity.^1,11,12,14^ Moreover, these channels can detect changes in membrane potential and open their conduction pathway as a result of membrane depolarization and subsequent conformational changes.^11^

The structure of H_V_1 consists only of a voltage-sensing domain (VSD) that contains four transmembrane segments (S1–S4). The S5–S6 pore-forming domain found in other voltage-gated ion channels is absent in H_V_1, resulting in the proton-selective permeation pathway being located within the S1–S4 transmembrane segments.^11^ In most species, the channel operates as a homodimer, and each monomer has its own voltage sensor, pH sensor, and proton permeation pathway and can function independently.^15,16^ Voltage sensitivity is conferred by the S4 segment, which contains three positively charged arginine residues, Arg205, Arg208, and Arg211.^2,12,17,18^ Upon membrane depolarization, these amino acid residues move outward, resulting in channel opening and proton conduction.^11,12,14^ In voltage-gated proton channels, only the open or closed states can be distinguished; there is no inactivation mechanism.^6,19^

H_V_1 channels are involved in many signaling pathways, and their most important role is the regulation of the intracellular pH.^6,7^ They normally conduct an outward current of H^+^ ions, driven by their electrochemical gradient.^11^ By controlling cytoplasmic pH, they are involved in many biological functions, such as the immune response and proliferation, motility and capacitation of human spermatozoa.^20^ Because H_V_1 activity is associated with NADPH oxidase (NOX)-dependent reactive oxygen species (ROS) production, these channels are also involved in neuroinflammation after brain injury and can promote cancer progression.^1,5^ Under physiological conditions, the channels are closed at resting membrane potential; however, under various pathological conditions (e.g., metabolic changes), they can open even at the resting membrane potential and contribute to the acidic cell microenvironment. Tumor cells are able to adapt extremely well to the acidic microenvironment, while immune cell functions are impaired.^21−24^ In fact, H_V_1 channel overexpression has been demonstrated in colorectal tumors, glioblastomas, and breast cancer, where these channels play an important role in migration, invasion and metastasis.^7,10,20,23,25−27^ In tumor samples from human patients, increased expression of H_V_1 correlated with poor disease prognosis.^24,26^ Overexpression of H_V_1 channels has been associated with disturbed pH balance and cancer development in several studies and has been proposed as a marker of malignancy in cancer.^11,23,27^

Inhibition of H_V_1 has been shown to reduce tumor cell proliferation, migration, along with cytokine and matrix metalloproteinase production.^23^ In breast cancer, xenotransplantation of H_V_1 knockout breast cancer cells into NOD/SCID-gamma (NSG) mice resulted in tumors with reduced sizes compared to tumors from mice transplanted with wild-type tumor cells.^26^

**Figure 1:**
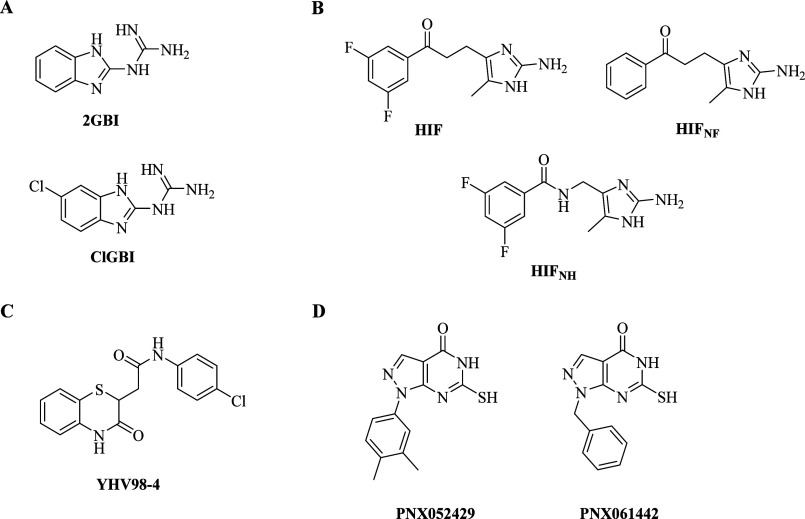
Structures of the known H_V_1 inhibitors. (A) Guanidine derivatives 2-guanidinobenzimidazole (2GBI) and 5-chloro-2-guanidinobenzimidazole (ClGBI);^30,31^ (B) “H_V_1 inhibitor flexible” compounds (HIFs);^32,33^ (C) YHV98–4;^5^ (D) PNX52429 and PNX61442.^24^

To date, insufficient structural information about this channel has hampered our understanding of the molecular mechanism of its activation and proton permeation and, in particular, the translation of this knowledge into specific H_V_1 inhibitors. Although numerous potential inhibitors have been investigated, H_V_1 inhibitors with good selectivity and an acceptable pharmacokinetic profile *in vivo* are still lacking.^1,28^ Several compounds have been identified as potential H_V_1 inhibitors in the past decade. The oldest and best known H_V_1 inhibitor is the zinc ion, which binds to two extracellular histidine residues, and acts as a competitor for protons. It stabilizes the resting state of the channel and its effect is detectable at micromolar concentrations.^1,2,24,28,29^ However, since Zn^2+^ is involved in various other physiological processes, its use as a specific H_V_1 channel blocker is limited.^1^ A series of guanidine derivatives has been described as reversible open-structure H_V_1 inhibitors. 2-Guanidinobenzimidazole (2GBI) and 5-chloro-2-guanidinobenzimidazole (ClGBI) (Figure 1A) are among the most effective H_V_1 inhibitors that bind to the intracellular side of the VSD.^30,31^ Furthermore, ClGBI was shown to block the channel even when applied in the extracellular medium.^31^ The so-called “H_V_1 inhibitor flexible” compounds (HIFs) (Figure 1B) were developed using structural modifications of guanidine derivatives to better explore the binding site for potential stabilizing interactions while reducing the overall hydrophobicity. Some of the HIF compounds have been reported to exhibit stronger inhibitory properties than guanidinobenzimidazoles.^32,33^ However, the selectivity of these compounds over other voltage-gated ion channels is low and their limited ability to penetrate the cell membrane makes them unsuitable for *in vivo* experiments.^1,20,34^ Recently, using a structure-based approach, YHV98–4 (Figure 1C) was identified as a selective voltage-gated proton channel inhibitor that binds to an intermediate conformational state of the protein, a transition state between the resting and activated states.^5^ By blocking H_V_1-mediated currents in DRG neurons, YHV98–4 has been shown to reduce chronic pain and, at micromolar concentrations, has an anti-inflammatory effect that may reduce morphine-induced adverse effects.^5^ Although the involvement of H_V_1 in neuronal excitability and as a potential analgesic target remains to be elucidated, given its good pharmacokinetic profile, YHV98–4 may prove to be a promising tool to explore the function of H_V_1 *in vitro* and *in vivo*. Recently, El Chemaly et al. discovered two new potential H_V_1 inhibitors, PNX52429 and PNX61442 (Figure 1D), by combining high-throughput screening (HTS) of a library of chemically diverse and commercially available compounds with compatible electrophysiological methods. These inhibitors provide potentially useful scaffolds with a favorable pharmacokinetic profile *in vitro* and *in vivo* that may be used to investigate the role of H_V_1 inhibitors as therapeutics for the treatment of tumors and inflammatory diseases.^24^

In this study, we describe the virtual-screening (VS)-based identification of a new structural class of small-molecule inhibitors of the human voltage-gated proton channel. For the screening, we used the 2GBI binding site of the open-conformation model of hH_V_1.^17^ We performed two separate VS campaigns, a screening of the commercial library and a screening of our in-house compound library. After inspection of results, the most promising hits were selected to be tested on hH_V_1 channels by patch-clamp electrophysiology.^17,31,32^ Based on the structures of the most promising inhibitors, additional compounds were selected for electrophysiological studies. In total, seven hits with a general 5-phenyl-2-aminoimidazole core were found to block the proton current in the low micromolar range and their antiproliferative activity was investigated in the cell lines MDA-MB-231 and THP-1. These molecules can be used as chemical tools to further understand the role of H_V_1 in tumors and other relevant diseases.

## Materials and Methods

### Structure-Based Virtual Screening

#### Compound Libraries Preparation

Fragment libraries from Asinex, Enamine, ChemBridge, Pharmeks, Life chemicals, Key Organics and Vitas-M were downloaded in SDF format. These libraries were merged, correct protonation states were assigned and duplicates removed, which resulted in a library of 118,570 compounds. In addition, an in-house library of 753 compounds was prepared in SDF format. For these compound libraries, an ensemble of conformers was generated with OMEGA software (Release 4.1.1.1, OpenEye Scientific Software, Inc., Santa Fe, NM, USA; www.eyesopen.com)^35^ using the default settings, which resulted in a maximum of 200 conformers per ligand.

#### Virtual Screening

A structural model of the open state of the human H_V_1 channel^17,29^ was used to perform virtual screening (VS) of the prepared multiconformer compound libraries. We performed two separate VS campaigns, a screening of the fragment-based commercial library and a screening of our in-house library of compounds. Both compound libraries were separately docked to the binding site of guanidine derivatives, which bind to the voltage-sensing domain of the H_V_1 channel from the intracellular side.^31^ The structure of human H_V_1 was generated using the Protein Preparation Wizard in Schrödinger Maestro Release 2022–1 (Schrödinger, LLC, New York, NY, USA, 2022) with the default settings: bond orders were assigned using the CCD database, missing hydrogen atoms were added, termini were capped, missing side chains were modeled using Prime, and het protonation states (pH 7.4) were modeled using Epik. The binding site for ligand docking was prepared using MAKE RECEPTOR (Release 4.2.1.1, OpenEye Scientific Software, Inc., Santa Fe, NM, USA; www.eyesopen.com). The grid box was centered on residues Asp112, Phe150, Ser181 and Arg211, which were shown to interact with the inhibitor 2GBI. The final grid box had the following dimensions: 20.67 Å * 17.00 Å * 19.33 Å and the volume of 6792 Å^3^. For “Cavity detection”, the slow and effective “Molecular” method was used for detection of binding sites. Two methods for detecting cavities are available in MAKE RECEPTOR. The Atomic and Molecular routines generate spatial representations or blobs in the 3D visualization that highlight the grooves and pockets surrounding the protein that can serve as potential binding sites. Although the Molecular detection method uses a superior algorithm, it requires significantly more time to execute compared to the Atomic detection algorithm. The inner and outer contours of the grid box were also calculated automatically using “Balanced” settings for “Site Shape Potential” calculation. There are three possibilities for calculating the Site Shape Potential. Selecting “Favors Protein” causes the contours to extend closer to the protein before extending into the solvent, while “Favors Solvent” has the opposite effect. In our case, the “Balanced” setting was used, which is reasonable in most cases. The inner contours were disabled. The side chain carboxylate group of Asp112 was defined as a hydrogen bond acceptor constraint for the docking calculations. The ligand structures were prepared with the LigPrep module and ionized with Epik at pH = 7.4 in Schrödinger Maestro Release 2022–1 (Schrödinger, LLC, New York, NY, USA, 2022). The small molecule library, prepared with OMEGA, was then docked to the prepared binding site using FRED available in OEDOCKING (Release 4.2.1.1. OpenEye Scientific Software, Inc., Santa Fe, NM, USA; www.eyesopen.com).^36,37^ The docking resolution was set to high, and other settings were set as default. A hit list of top 1000 ranked molecules from a commercial compound library and a hit list of all molecules from the in-house compound library were retrieved and the best ranked FRED-calculated pose for top 100 ranked compounds from each library was visually inspected and used for analysis. From the highest ranked compounds, 10 were selected from the in-house library and 10 were selected and purchased from Enamine Ltd. to test their inhibitory activity by patch-clamp electrophysiology on cell lines expressing H_V_1 channels.

### Chemistry

#### Synthesis

The synthesis and characterization of **13**, **19**, **21**–**29**, **31**–**36**, and **38**–**41** have been previously reported.^38−41^ Synthesis and characterization data of compounds **30**, **37**, **42**, **43** and 5-chloro-2-guanidinobenzimidazole (ClGBI) can be found in the Supporting Information.

#### Materials

The reagents and solvents used were obtained from commercial sources (i.e., Acros Organics, Sigma-Aldrich, TCI Europe, Merck, Carlo Erba, Apollo Scientific, Fluorochem, Enamine) and were used as provided. Analytical thin-layer chromatography (TLC) was performed on silica gel aluminum sheets (60 F254, 0.20 mm; supplied by Merck) under visualization with UV light and spray reagents. Flash column chromatography was performed using Kieselgel 60, with a granulometry of 0.040–0.063 nm (230–400 mesh ASTM), supplied by Merck, as stationary phase. ^1^H and ^13^C NMR spectra were recorded at 400 and 100 MHz, respectively, on a Bruker Avance III NMR spectrometer (Bruker, MA, USA) at 295 K. The chemical shifts (δ) are reported in ppm and are referenced to the deuterated solvent used (CDCl_3_ or in DMSO-*d*_*6*_, according to the solubility of each compound), with tetramethylsilane (TMS) as internal standard. Mass spectrometry (MS) measurements were performed on an Expression CMS^L^ mass spectrometer (Advion, NY, USA). High resolution mass spectrometry (HRMS) measurements were performed on a HPLC–MS/MS system (Q Executive Plus; Thermo Scientific, MA, USA). Analytical reversed-phase UPLC analyses were performed using a modular system (Thermo Scientific Dionex UltiMate 3000 modular system; Thermo Fisher Scientific Inc., MA, USA). Method: Waters Acquity UPLC HSS C18 SB column (2.1 × 50 mm, 1.8 μm), T = 40 °C; injection volume = 1.750 μL (0.20–0.30 mg of sample, dissolved in 100 μL of DMSO and 900 μL of MeOH); flow rate = 0.3 mL/min; detector λ = 254 and 280 nm; mobile phase A (0.1% trifluoroacetic acid (TFA) [v/v] in water), mobile phase B methanol (MeOH). Gradient: 0–8 min, 10–90% B; 8–10 min, 90% B; 10–11 min, 90–10% B. Purities of the tested compounds were established to be ≥95% at 254 and 280 nm, as determined by UPLC.

The structures were drawn with ChemDraw 20.0 (PerkinElmer), the NMR spectra were analyzed with MestReNova v12.0.0–20080 (© 2017 Mestrelab Research S.L.) and the HPLC-MS spectra were analyzed with Advion Data Express v6.4.10.3.

### Biological Evaluation

The selected VS hits and the prepared and characterized compounds were evaluated for their inhibitory activity by patch-clamp electrophysiology on reporter cell lines expressing H_V_1 channels.

#### Cells for Patch Clamp Recordings

Chinese hamster ovary (CHO) cells were cultured in Dulbecco’s modified Eagle’s medium (DMEM, Gibco, Thermo Fisher Scientific, Waltham, MA, USA, Cat# 11965084) containing 10% fetal bovine serum (FBS, Sigma-Aldrich), 2 mM l-glutamine, 100 μg/mL streptomycin, and 100 U/mL penicillin-g (Sigma-Aldrich, St. Louis, MO, USA) at 37 °C in a 5% CO_2_ and 95% air humidified atmosphere. Cells were passaged twice per week following a 2–3 min incubation in PBS containing 0.2 g EDTA/L (Invitrogen, Waltham, MA, USA). For the patch-clamp experiments, CHO cells were carefully washed twice with 2 mL of ECS (see below). CHO cells were transiently transfected using a Lipofectamine 2000 kit (Invitrogen, Carlsbad, CA, USA) according to the manufacturer’s protocol with a pQBI25 vector containing the GFP-tagged hH_V_1 gene (*hHVCN1*, GenBank accession no. BC007277.1, a kind donation from Kenton Swartz, NIH, Bethesda, MD, USA). Transfected cells were washed twice with 2 mL of ECS (see below) and replated onto 35 mm polystyrene cell culture dishes (Cellstar, Greiner Bio-One, Kremsmünster, Austria). GFP-positive transfectants were identified with a Nikon Eclipse TS100 fluorescence microscope (Nikon, Tokyo, Japan) using bandpass filters of 455–495 nm and 515–555 nm for excitation and emission, respectively, and were used for current recordings. In general, ion currents were recorded 24 to 36 h after transfection.

#### Electrophysiology

The standard whole-cell patch clamp method^42^ was used to record the ion currents. Micropipettes were pulled in four stages using a Flaming Brown automatic pipet puller (Sutter Instruments, San Rafael, CA, USA) from GC 150F-15 borosilicate glass capillaries (Harvard Apparatus Co., Holliston, MA, USA) with a typical tip resistance between 2 and 8 MΩ. All measurements were performed using Axopatch 200B amplifiers connected to personal computers with Digidata 1550A data acquisition hardware (Molecular Devices Inc., Sunnyvale, CA, USA). In general, the holding potential was −60 mV. Recordings were discarded if a leak at the holding potential was more than 10% of the peak current at the given test potential. The experiments were conducted at room temperature, which was between 20 and 24 °C.

#### Solutions

The extracellular (bath) solution (ECS) contained (in mM) 75 *N*-methyl d-glucamine (NMDG), 180 HEPES, 15 glucose, 3 MgCl_2_, and 1 EGTA (pH = 7.4 with CsOH), and the intracellular (pipet) solution (ICS) contained (in mM) 75 NMDG, 180 MES, 3 MgCl_2_, 15 glucose, and 1 EGTA (pH = 6.4 with CsOH). The osmolarities of ECS and ICS were between 302 and 308 mOsm/L and ∼295 mOsm/L, respectively. Bath perfusion around the measured cell with different extracellular solutions was achieved using a gravity flow microperfusion system at a rate of 200 μL/min. Excess fluid was removed continuously. Solutions containing the test compounds were made fresh before the experiments in ECS from 10 mM stock solutions stored at −20 °C. Stock solutions were prepared from powder dissolved in anhydrous DMSO (Sigma-Aldrich Hungary). The control solution was ECS with 0.5% DMSO. ECS with a pH of 6.4 was used as a perfusion test for each cell. The reduction in the current amplitude and the prominent change in the current activation threshold were indicators of both the ion channel and the proper operation of the perfusion system.

#### Voltage Protocols

The current through hH_V_1 was elicited by applying a 1.0-s-long voltage ramps to +60 mV or +100 mV from a V_h_ of −60 mV every 15 s.

#### Patch Clamp Data Analysis

The pClamp 10.7 software package (Molecular Devices Inc., Sunnyvale, CA, USA) and GraphPad Prism 8 (GraphPad, CA, USA) were used for data acquisition and analysis. The H_V_1 current recordings were evaluated as follows. First, the traces were filtered (lowpass boxcar, 3 smoothing points), and off-line leaks were corrected. As leak is an ohmic current (i.e., the voltage–current relationship is linear), we defined a region in which the opening probability of the H_V_1 channels is approximately zero. Thus, a linear regression line was fitted to the data points between 16 and 80 ms, corresponding to −60 mV and −53 mV, and the fitted parameters were used to subtract the nonspecific leak. The leak-corrected currents between +59 mV and +60 mV were extracted, averaged, and considered as the peak current.

The inhibitory effect of the compounds at a given concentration was calculated as the remaining current fraction (*RCF* = I/I_0_, where I_0_ is the peak current in the absence of the compound, and I is the peak current at equilibrium block at 50 μM concentration of the compound). The data points (average of 3–5 individual recordings) in the dose–response curve were fitted with a two-parameter inhibitor vs response model using the following formula
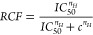
where *c* is the molar concentration of the compound, IC_50_ is the concentration of the compound that inhibits the channel current by 50%, and *n*_*H*_ is the Hill coefficient. All data are expressed as mean ± SEM.

#### Cytotoxicity Measurements

The antiproliferative effect of the selected compounds was investigated in the triple negative breast cancer cell line MDA-MB-231 (ATCC HTB-26) derived from a 51-year-old Caucasian woman and human monocytic leukemia cell line THP-1 isolated from an acute monocytic leukemia patient. An MTS (3-(4,5-dimethylthiazol-2-yl)-5-(3-carboxymethoxyphenyl)-2-(4-sulfophenyl)-2*H*-tetrazolium) assay was performed according to the manufacturer’s instructions. MDA-MB-231 and THP-1 cells were procured from the American Type Culture Collection (ATCC, Manassas, VA, USA) and cultured in Roswell Park Memorial Institute (RPMI) 1640 medium with HEPES (Sigma-Aldrich, St. Louis, MO, USA). The culture medium contained 10% fetal bovine serum (Gibco, Thermo Fisher Scientific, Waltham, MA, USA), 100 U/mL penicillin, 100 μg/mL streptomycin, and 2 mM l-glutamine. MDA-MB-231cells were seeded in 96-well plates at 3000 cells per well and incubated overnight in a 5% CO_2_ atmosphere at 37 °C. The following day, cells were treated with the specified compounds, positive control (17-DMAG, a known Hsp90 inhibitor) or vehicle control (0.5% DMSO). THP-1 cells were seeded in 96-well plates at 20000 cells per well and then treated with the specified compounds, positive control (PU-H71, a known Hsp90 inhibitor) or vehicle control (0.5% DMSO). Both MDA-MB-231 and THP-1 cell were then incubated for 72 h in a 5% CO_2_ atmosphere at 37 °C. Subsequently, 10 μL of CellTiter96 Aqueous One Solution Reagent (Promega, Madison, WI, USA) was added to each well, and the cells were incubated for a further 3 h. Absorbance was measured at 492 nm using a microplate reader (Synergy 4 Hybrid; BioTek, Winooski, VT, USA). Two independent experiments, each performed in triplicate, were conducted. Statistical significance (*p* < 0.05) was determined through a two-tailed Welch’s *t* test, comparing the treated groups with DMSO. IC_50_ values, denoting the concentration at which a compound induces a half-maximal response, were calculated using GraphPad Prism 10.0 software (San Diego, CA, USA) and are presented as averages from independent measurements.

## Results and Discussion

### Structure-Based Virtual Screening

Given the lack of potent and selective compounds to study H_V_1 hyperactivity, we pursued a structure-based hit identification project to discover a novel structural class of H_V_1 inhibitors. We performed a virtual screening (VS) using a structural model of the open state of the human H_V_1 channel^17^ and the proposed binding site of one of the best-known Hv1 inhibitors 2GBI.^17,30,33^ To validate our docking protocol, we included 2GBI in our study. The binding mode of 2GBI was well reproduced, involving stabilizing interactions with Asp112 and Asp185 (Figure S1; Supporting Information).^31,33^

Prior to structure-based VS, we prepared two separate multiconformer compound libraries. The first library contained more than 118,000 commercially available fragment-like compounds, while the second contained our 753 in-house compounds. A library of small fragments was used because most of the known H_V_1 inhibitors have a relatively low molecular weight, and because smaller molecules are more suitable for potential subsequent medicinal chemistry optimization. All compounds were docked to the proposed binding site of guanidine derivatives, which bind to the voltage-sensing domain of the H_V_1 channel from the intracellular side.^31^ Docking calculations were performed using FRED as available in OEDOCKING (Release 4.2.1.1. OpenEye Scientific Software, Inc., Santa Fe, NM, USA; www.eyesopen.com).^36,37^ The compounds were ranked according to the Chemgauss4 score of the best ranked conformation. The results of the VS were carefully examined and the predicted binding modes of the 100 highest-ranked compounds were visually inspected.

According to the predicted binding modes, the highest-ranked ligands frequently formed interactions with Asp112 in the S1 helix (result of employing the hydrogen bond constraint in the docking algorithm), which is crucial for the inhibition of proton permeation, and with Phe150 and Asp185 in helices S2 and S3, respectively. Occasionally, they formed interactions with Glu153 in the S2 helix and Arg211 in the S4 helix and fitted well into the pocket defined by Asp112, Phe150, Ser181 and Arg211.^31^ Many of these residues are also involved in the binding of 2GBI and its analogs.^31,33^ Hydrophobic interactions with the side chains of Ile144, Ile146 and Leu147 in the S2 helix and Ile183, Leu184 and Ile186 in the S3 helix often contributed to the stabilization of the ligand-protein complexes. The compounds were selected for biological evaluation based on their scoring function values, favoring compounds that had similar pharmacophore groups to the guanidine derivatives and resembled their binding modes.^31,33^ To cover a broad structural diversity, only one representative from each structural class was included.

Based on the structural differences and predicted binding modes, ten compounds from the commercial library (Table 1), and ten compounds from our in-house library (Table 2) were selected for biological evaluation.

**Table 1:**
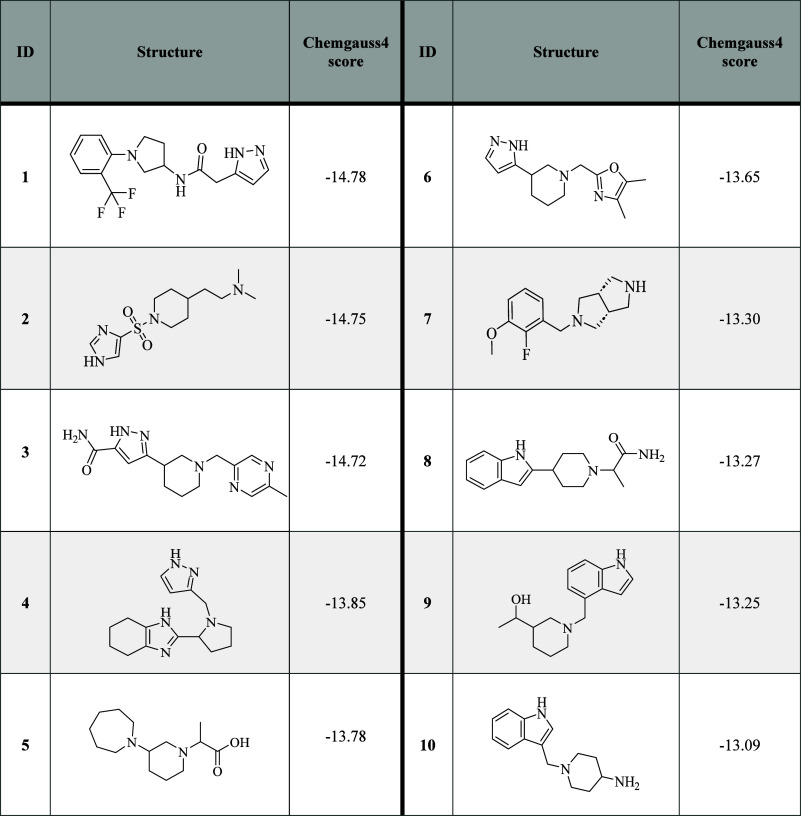
Commercial Fragment-Based Library: Structures and Predicted Binding Free Energies of the Selected Virtual Screening Hits

**Table 2:**
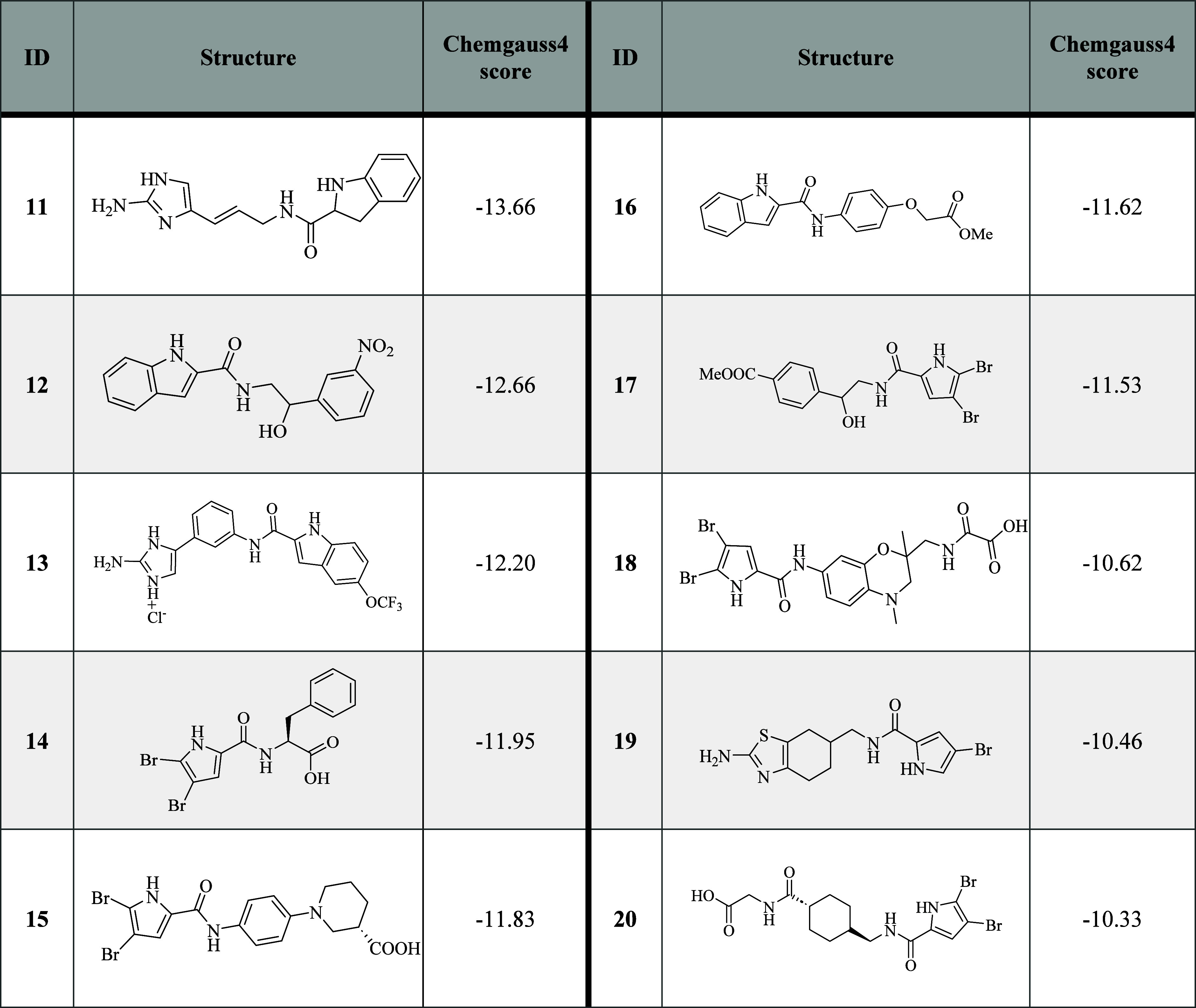
In-House Library: Structures and Predicted Binding Free Energies of the Selected Virtual Screening Hits

### Biological Evaluation

#### Evaluation of the Compounds from VS Campaigns

Ten compounds from the commercial fragment-based library and ten compounds from the in-house library were tested for their inhibitory effects on human voltage-gated proton channels. The selected compounds were tested by manual patch-clamp technique on Chinese hamster ovary (CHO) cells transiently expressing hH_V_1 at a concentration of 50 μM. We used voltage ramps to record whole-cell currents since in addition to the current amplitude measured at the highest applied voltage, the opening threshold of the channels is also readily measurable. This is informative as certain compounds may shift the voltage-dependence of H_V_1 gating. Proper functioning of the perfusion apparatus was confirmed using ECS at a pH of 6.4, which significantly shifts the opening threshold of the channel toward positive membrane potentials, as the gating of H_V_1 depends on both the transmembrane pH gradient and the membrane potential. Moreover, to rule out possible artifacts due to pH-shifts caused by proton accumulation we have done several measures: 1. All solutions were heavily pH-buffered. 2. Patch-clamped cells were continuously perfused with external solution preventing the local accumulation of protons due to the outward currents. 3. Measurements were only started after the currents had stabilized, i.e. several sequential traces were practically overlapping, thus ruling out the possibility of H^+^ accumulation. In addition, none of the compounds contains strong acidic or basic centers that would be able to significantly alter the local pH. Our preliminary results showed that none of the purchased compounds exhibited channel inhibition at 50 μM (Table 3). RCF values represent the remaining H_V_1 current fraction measured at +60 mV in the presence of 50 μM of the compounds.

**Table 3:** H_V_1 Inhibitory Potencies of 10 Compounds from the Commercial Fragment-Based Library

ID	RCF^a^	*n*^b^	ID	RCF	*n*
**1**	1.04 ± 0.09	2	**6**	1.12 ± 0.15	2
**2**	1.08 ± 0.13	3	**7**	1.02 ± 0.10	3
**3**	1.09 ± 0.19	2	**8**	1.09 ± 0.07	3
**4**	1.09 ± 0.15	2	**9**	0.97 ± 0.08	3
**5**	1.05 ± 0.08	3	**10**	1.35 ± 0.17	2

a Remaining H_V_1 current fraction (RCF) measured at +60 mV in the presence of 50 μM of the compounds. All data are presented as means ± SEM.

b Sample size. ClGBI was used as a positive control. IC_50_ value for ClGBI was determined to be 15.9 ± 2.0 μM.

**Figure 2:**
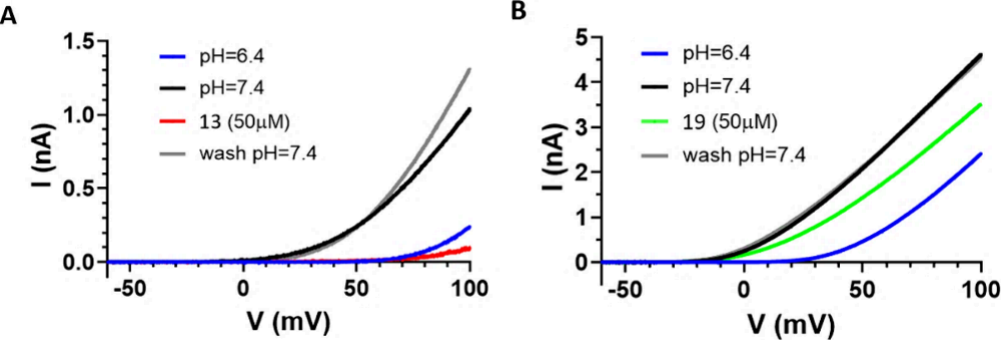
Inhibition of hH_V_1 currents by **13** (A) and **19** (B). Peak currents were determined as the current amplitudes at the end of the ramps at the highest applied voltage. The details of the protocols and experimental conditions are described in Materials and Methods.

**Figure 3:**
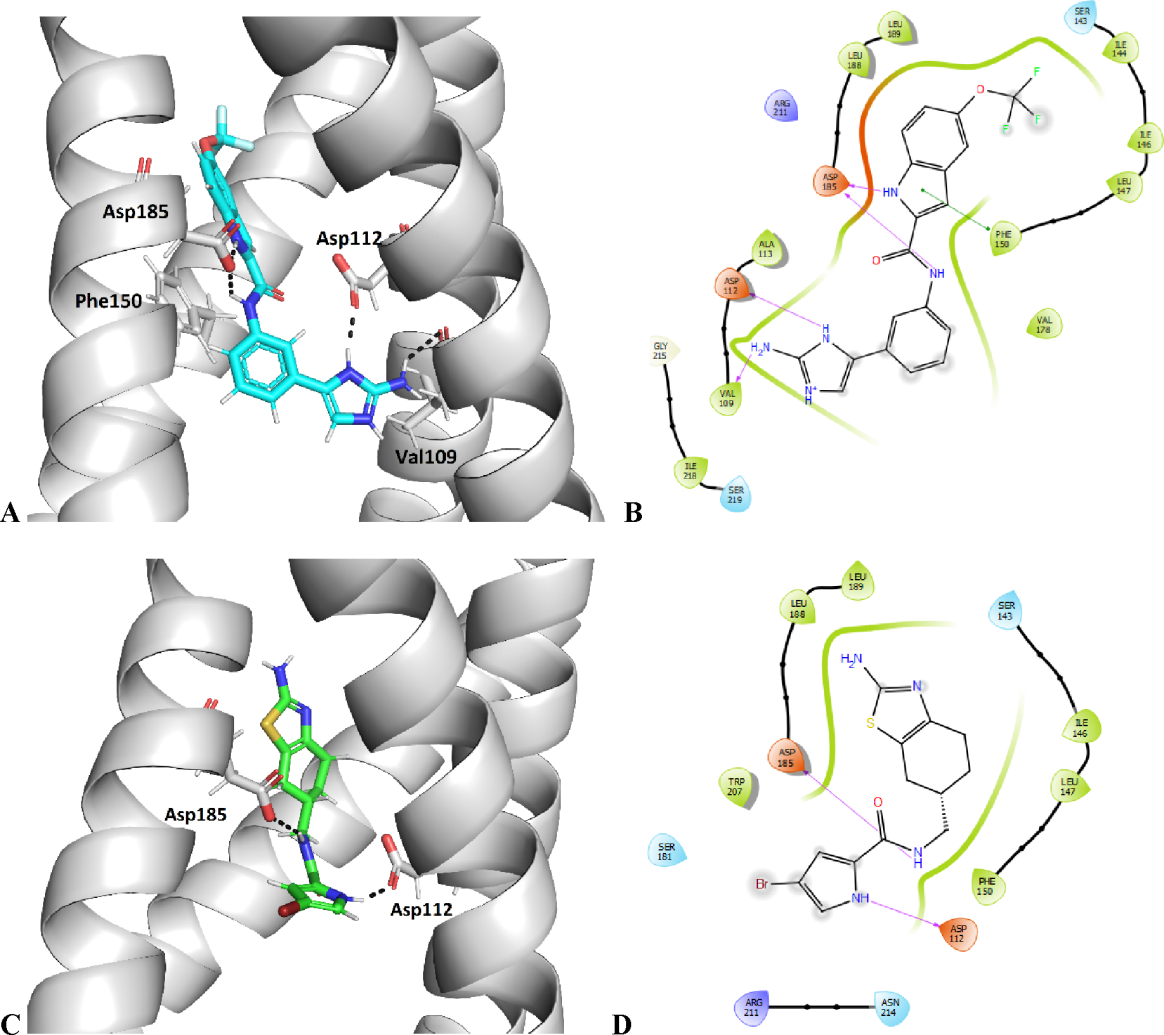
Binding mode of **13** (A, B) and **19** (C, D) in the proposed binding pocket in the H_V_1 VSD, predicted by docking with FRED. The ligand and the neighboring protein side chains are shown as stick models, colored according to the chemical atom type (blue, N; red, O; green, F). For clarity, only key amino acids are shown. H-bonds are indicated by black dotted lines. In the schematic representations of protein–ligand interactions in B and D, the hydrogen bonds are shown as arrows (in magenta), the π-stacking is shown as green line and the hydrophobic contacts are indicated as gray spheres.

More promising results were obtained from the evaluation of the ten compounds selected from our in-house library (Table 4). We identified two compounds that significantly decreased the proton flux through the H_V_1 channel at 50 μM. A strong inhibition was demonstrated by the 2-aminoimidazole derivative **13** that showed an approximately 80% reversible decrease in proton current at 50 μM (RCF value = 0.19) (Table 4; Figure 2A; Figures S2, S3; Supporting Information). An illustration of the current amplitudes and opening threshold potential of the channels showing the tail currents at the end of the ramp protocols was included in Figure S4 of the Supporting Information. The docking predicted that the N-1 nitrogen and the 2-amino group of the 2-aminoimidazole ring of **13** were within the hydrogen bonding distance of Asp112 and Val109, respectively. Additional hydrogen bonds were formed between the Asp185 side chain and the amine groups of the indole ring and the amide bond. The benzene ring of the indole moiety was further stabilized by π-stacking interactions with Phe150. Several additional hydrophobic contacts were formed between the amino acid residues of H_V_1 and inhibitor **13** (Figure 3 A, B). The 4,5,6,7-tetrahydrobenzo[*d*]thiazole derivative **19** showed a reversible reduction in proton current of about 30% at a concentration of 50 μM (RCF value = 0.71). The H_V_1 current traces measured in the presence of **19** are shown in Figure 2B. Compound **19** was predicted to form hydrogen bonds with Asp112 and Asp185 side chains and was further stabilized in the binding site by several hydrophobic interactions (Figure 3 C, D).

**Table 4:** H_V_1 Inhibitory Potencies of 10 VS Hits Selected from In-House Library

ID	RCF^a^	IC_50_ (μM)^b^	*n*^c^	ID	RCF	IC_50_ (μM)	*n*
**11**	0.98 ± 0.04	n.d.^d^	2	**16**	0.96 ± 0.07	n.d.	2
**12**	0.96 ± 0.01	n.d.	2	**17**	1.07 ± 0.04	n.d.	2
**13**	0.19 ± 0.11	8.5 ± 0.6	5	**18**	0.98 ± 0.04	n.d.	2
**14**	0.90 ± 0.02	n.d.	2	**19**	0.79 ± 0.05	n.d.	3
**15**	0.96 ± 0.06	n.d.	2	**20**	1.03 ± 0.02	n.d.	2

a Remaining H_V_1 current fraction (RCF) measured at +60 mV in the presence of 50 μM of the compounds.

b Half maximal inhibitory concentration. All data are presented as means ± SEM.

c Sample size

d n.d. – not determined. ClGBI was used as a positive control. IC_50_ value for ClGBI was determined to be 15.9 ± 2.0 μM.

#### Analogs of the Hit Compound **13** and Structure–Activity Relationships

Our initial results highlighted compound **13** as a promising new H_V_1 inhibitor, therefore we decided to investigate this structure further. In our in-house library, we identified an additional 23 compounds with a 5-phenyl-2-aminoimidazole or 5-phenyl-4,5-dihydro-2-aminoimidazole scaffold and investigated their effects on H_V_1 channels by electrophysiology (**21**–**43**, Table 5). All of these compounds were included in the initial VS, and most of them were among the highest ranked compounds and assumed similar orientations to **13** in the binding pocket.

**Table 5:**
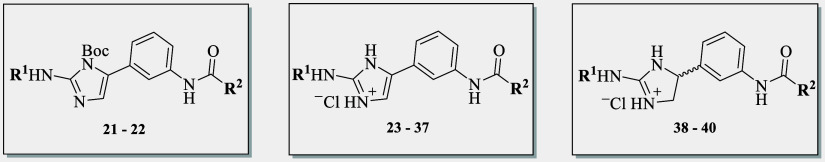

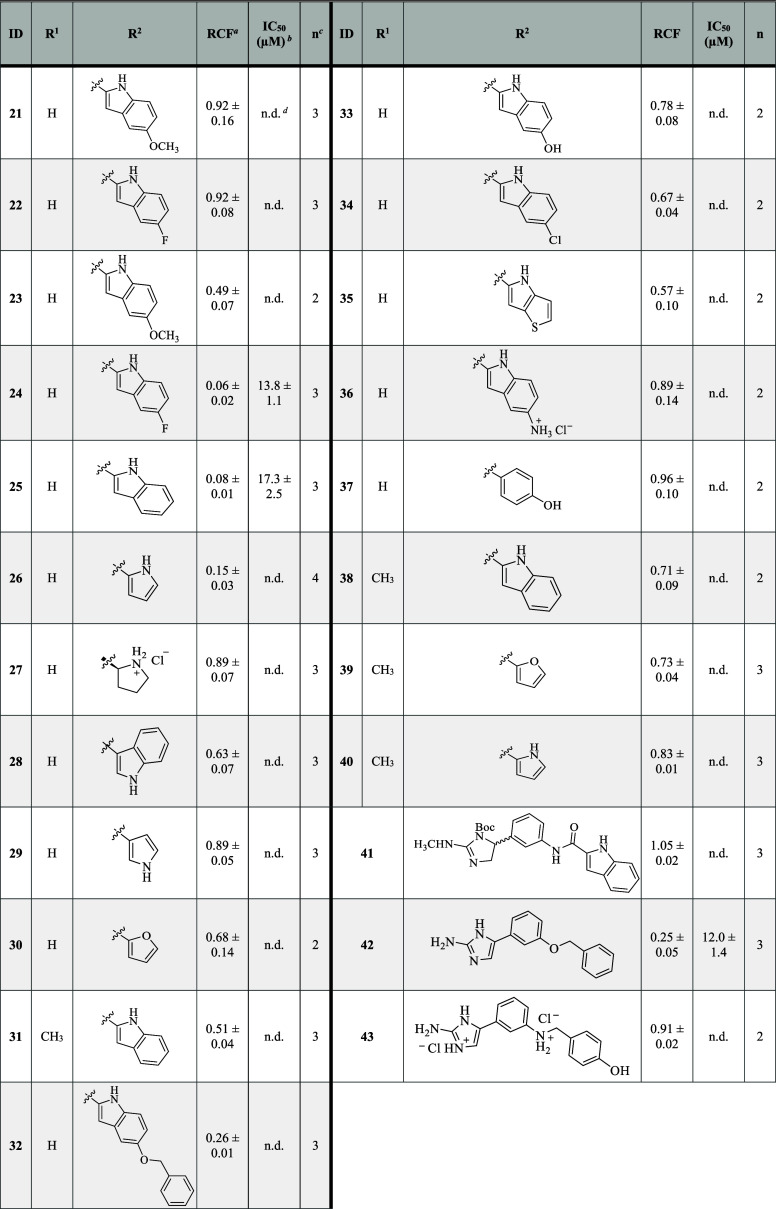
H_V_1 Inhibitory Effects of 5-Phenyl-2-aminoimidazoles **21**–**37**, **42** and **43** and 5-Phenyl-4,5-dihydro-2-aminoimidazoles **38**–**41** Selected from the In-House Library

a Remaining H_V_1 current fraction (RCF) measured at +60 mV in the presence of 50 μM of the compounds.

b Half maximal inhibitory concentration.

c Sample size.

d n.d. – not determined. ClGBI was used as a positive control. IC_50_ value for ClGBI was determined to be 15.9 ± 2.0 μM.

Of the 23 new compounds tested, 16 contain a 2-aminoimidazole ring (**23**–**30**, **32**–**37**, **42** and **43**). This moiety has already been recognized as an H_V_1 arginine mimic and is also present in the structure of the hit **13**.^30,32^ Compounds **31** and **38**–**41** have an additional methyl substituent on the 2-amino group of the 2-aminoimidazole ring, while **21**, **22** and **41** have a *tert*-butyloxycarbonyl (Boc) substituent on the N-1 imidazole nitrogen. Four 4,5-dihydro-2-aminoimidazoles (**38**–**41**) with a reduced imidazole C = C bond were also investigated. In all molecules, a central benzene ring is present, which is linked to substituted aromatic or aliphatic groups at position 3. In most compounds, these groups are connected to the central benzene ring by an amide bond, while compounds **42** and **43** contain a more flexible oxymethylene or aminomethylene linker at this position.

Six compounds (**23**–**26**, **32** and **42**) inhibited the channel function by more than 50% at 50 μM, five compounds (**28**, **30**, **31**, **34** and **35**) showed weak channel block, with RCF values between 0.50 and 0.70, while the remaining 12 compounds were inactive. Of the six most active compounds, **42** is structurally the most different from **13** as it contains a benzyloxy group attached to the central benzene ring. The high activity of this analog (RCF = 0.25) indicates that nitrogen-containing heterocycles, such as indole or pyrrole rings, are not essential for channel inhibition. Additionally, the results show that the amide bond connecting the central benzene ring to the aromatic groups can be replaced by an oxymethylene linker. In general, the introduction of an *N*-methyl group at the primary amino group of the 2-aminoimidazole ring results in lower activity, as shown by a comparison of compound **25** (RCF = 0.08) with its *N*-methylated analog **31** (RCF = 0.61). A similar decrease in activity was observed when the Boc substituent was introduced at the N-1 nitrogen of the imidazole (comparison of compounds **21** and **22** with **23** and **24**). These results indicate the importance of the free primary amino group at position 2 and the absence of substituents at the N-1 nitrogen. The impaired binding to the channel caused by the introduction of hydrophobic substituents at the imidazole nitrogen is consistent with previous studies investigating the molecular properties of guanidine-based H_V_1 inhibitors.^31^ Moreover, the activity is completely lost when the C = C bond of the imidazole is reduced, as in the case with compounds **38**–**41**. This leads to decreased planarity of the molecules, which could be unfavorable for binding. In general, small changes in aromatic groups at position 3 of the central benzene ring had a large effect on the activity. Replacing the indole-2-carboxamide group (**25**, RCF = 0.08) with the indole-3-carboxamide (**28**, RCF = 0.63) resulted in a significant decrease in activity. Similarly, the activity decreased when the indole ring was replaced by a thieno[3,2-*b*]pyrrole ring (**35**, RCF = 0.57) or when the pyrrole ring (**26**, RCF = 0.15) was replaced by the furan ring (**30**, RCF = 0.68). The presence of a polar substituent at position 5 of the indole ring, such as amine (**36**, RCF = 0.89) and hydroxyl groups (**33**, RCF = 0.78), resulted in inactive compounds. Similar was also observed for compound **37** with a 4-hydroxyphenyl group (RCF = 0.96) and for compound **27** with the basic pyrrolidine group (RCF = 0.89). Some of the most potent compounds in the series contained either an unsubstituted indole (**25**), or 5-substituted indole groups, such as 5-fluoroindole (**24**), 5-trifluoromethylindole (**23**), or 5-benzyloxyindole (**32**). Interestingly, the potent activity of **32** (RCF = 0.26) with the large benzyloxy substituent suggests that there is sufficient space in the binding pocket for larger lipophilic groups in this position.

**Figure 4:**
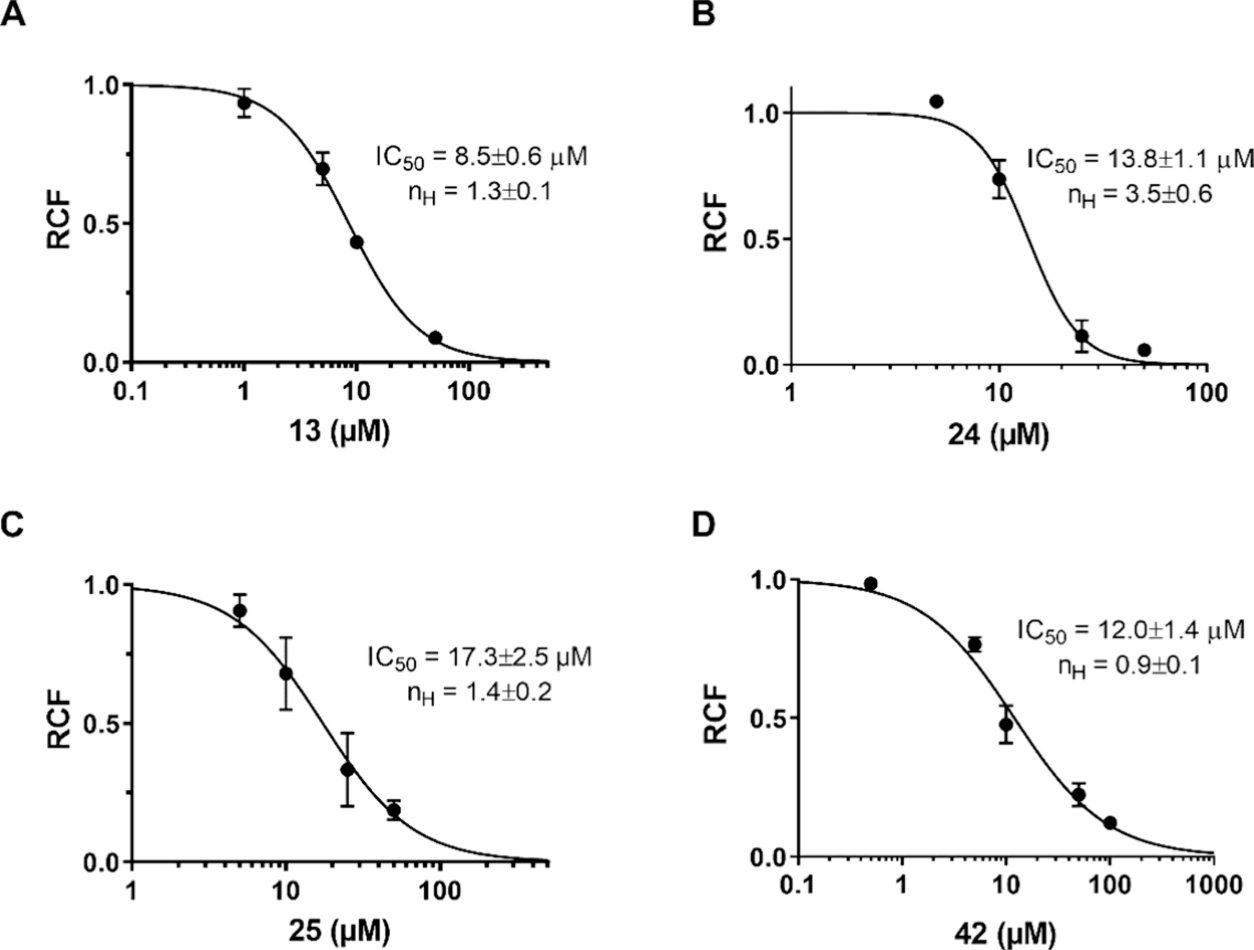
Concentration-dependent inhibition of the H_V_1 channel by **13** (A), **24** (B), **25** (C) and **42** (D). Points on the dose–response curves represent the mean of 4–5 independent measurements. Data points were fitted with a two-parameter Hill equation (see Materials and Methods). The best fit yielded IC_50_ values of 8.5 ± 0.6 μM for **13**, IC_50_ = 13.6 ± 1.1 μM for **24**, IC_50_ = 17.3 ± 2.5 μM for **25** and IC_50_ = 12.0 ± 1.4 μM for **42** (*n* ≥ 3 for all concentrations). Hill coefficient is labeled as n_H_. Error bars represent SEM.

For compounds **13**, **24**, **25**, and **42**, dose–response experiments were performed (Figure 4). Different concentrations of the molecules were applied to the cells to reach the equilibrium block. The RCF values were calculated and plotted as a function of compound concentration. The measured IC_50_ values were 8.5 ± 0.6 μM for **13**, 13.8 ± 1.1 μM for **24**, 17.3 ± 2.5 μM for **25** and 12.0 ± 1.4 μM for **42** (*n* ≥ 3 for all concentrations), confirming the concentration-dependent inhibition of the H_V_1 channel. The inhibitory activities were even better than those reported for 2GBI, ClGBI and HIF, with IC_50_ values of 38.3 ± 0.7 μM, 26.3 ± 2.2 μM and 13.3 ± 1.0 μM, respectively.^30−32^

To investigate the role of the phenyl ring in H_V_1 binding, compound **44**, an analogue of **25** with an alkyl chain replacing the central phenyl ring, was additionally investigated. The results are shown in Figure S5 of the Supporting Information. As can be seen from Figure S5, compound **44** does not show good inhibitory activity on H_V_1 channels, with an RCF value of 0.79 at a concentration of 50 μM. This indicates that the central phenyl ring is crucial for the high binding affinity of this class of H_V_1 inhibitors.

Of the seven most potent compounds that inhibited H_V_1 channel function by more than 50% at 50 μM, two, **25** and **26**, have previously been evaluated for their activity on human voltage-gated sodium channel (Na_V_) isoforms.^40,43^ Compound **25** showed moderate activity on Na_V_1.2, Na_V_1.4, Na_V_1.5, and Na_V_1.6 at 10 μM, but no effect on Na_V_1.3, Na_V_1.7 and Na_V_1.8.^40,43^ Interestingly, compound **26**, one of the most potent compounds in the series, had no effect on any of the Na_V_ isoforms tested at up to 30 μM.^40^

#### Cytotoxicity Measurements

**Figure 5:**
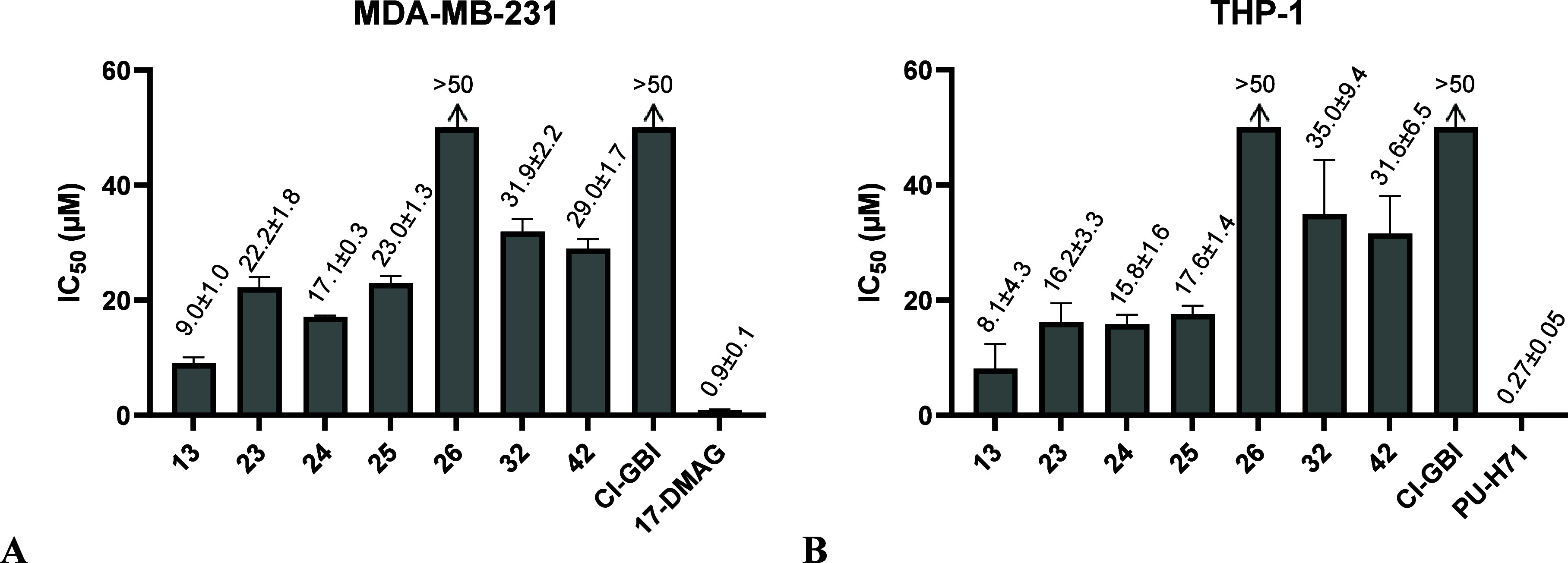
Graphical representation of the antiproliferative IC_50_ values determined for all compounds that inhibit H_V_1 function by more than 50% at 50 μM and for ClGBI in the breast cancer cell line MDA-MB-231 (A) and in the human monocytic leukemia cell line THP-1 (B). 17-DMAG and PU-H71, known Hsp90 inhibitors, were used as positive controls. Data represent mean ± SD of at least two independent experiments performed in triplicate.

The cytotoxic activities of eight H_V_1 inhibitors (**13**, **23**–**25**, **31**, **32**, **34** and **35**) against the hepatocellular carcinoma cell line Huh-7 have been reported previously.^41^ In general, the highest activities (EC_50_; 20 to 50 μM) were obtained for compounds that showed the strongest effect on H_V_1 channels. Some of the compounds were also previously evaluated against human hepatocellular carcinoma HepG2 and acute monocytic leukemia THP-1 cell lines.^44^ Interestingly, the active compounds **13**, **23**–**26**, **28**, **31**, **32**, **34** and **35** showed moderate to strong apoptosis-inducing activity with EC_50_ values between 13 and 42 μM, while the inactive compounds **27**, **29**, **33**, **39** and **40** showed only weak apoptosis-inducing activity with 16–28% apoptotic cells at 50 μM.^44^ To further investigate the antiproliferative effect of our H_V_1 inhibitors, we tested all compounds that inhibited H_V_1 function by more than 50% at 50 μM (**13**, **23**–**26**, **32** and **42**) by MTS assay in two additional cell lines, the triple negative breast cancer cell line MDA-MB-231 and the human monocytic leukemia cell line THP-1. It has previously been demonstrated that H_V_1 is highly expressed in the metastatic human breast cancer cell line MDA-MB-231, promoting invasion and metastasis.^23,26,27^ The expression of H_V_1 in the THP-1 cell line is even higher than in MDA-MB-231, and the H^+^ current has been detected and analyzed in detail in this cell line.^45^ The measured IC_50_ values are presented graphically in Figure 5A and 5B. The IC_50_ values and dose–response curves can be found in Tables S1 and S2, and Figures S6 and S7, respectively (Supporting Information). The strongest growth inhibitor of the set was compound **13** (MDA-MB-231 IC_50_ = 9.0 ± 1.0 μM, THP-1 IC_50_ = 8.1 ± 4.3 μM), which also showed the strongest inhibition of the H_V_1 channel (IC_50_ = 8.5 ± 0.6 μM), followed by compound **24** (MDA-MB-231 IC_50_ = 17.1 ± 0.3 μM, THP-1 IC_50_ = 15.8 ± 1.6 μM), which had the lowest RCF value (0.06). Surprisingly, compound **26** was not able to reduce the growth of cancer cells, as was the case with ClGBI. A possible factor could be insufficient membrane permeability. ClGBI contains a guanidinium group in its structure, which is positively charged under physiological conditions and could limit membrane permeability.^31^ Overall, the *in vitro* evaluation on MDA-MB-231 and THP-1 cell lines demonstrates the anticancer potential of the identified new hH_V_1 inhibitors.

## Conclusions

In summary, we used computational methods to discover the 5-phenyl-2-aminoimidazoles as a new structural class of inhibitors of human voltage-gated proton channels H_V_1. Altogether 43 compounds were identified by structure-based virtual screening on an open structure of the human H_V_1 channel. The compounds were tested on CHO cell lines transiently expressing hH_V_1, and the most promising inhibitors showed IC_50_ values in the low micromolar range. The obtained results enabled us to investigate the structure–activity relationship of this new structural class of H_V_1 inhibitors. The unsubstituted 2-aminoimidazole ring proved to be the most important part of the molecules for a good inhibitory effect on H_V_1. Some of the most potent inhibitors contained an unsubstituted or 5-substituted indole-2-carboxamide group on the right-hand side. Various modifications were tolerated at this position, which appears to be the most suitable for further optimization. Given the relatively small structure of the 5-phenyl-2-aminoimidazole scaffold, it offers great potential for the introduction of modifications to enhance the inhibitory activity on hH_V_1, reduce potential off-target effects and improve physicochemical properties. Considering the lack of potent and selective inhibitors of H_V_1 channels, the current study represents a solid starting point for further research in the field of H_V_1 inhibitors, which have the potential to be developed into therapeutically useful agents for the treatment of various diseases. In addition, the H_V_1 inhibitors we discovered in this work could be used as tools to study the biological role of this channel.

## Data Availability

The following third-party software was used. The compound libraries were prepared with OMEGA software, version 4.1.1.1 (OpenEye Scientific; www.eyesopen.com). The binding site for ligand docking was prepared using MAKE RECEPTOR, version 4.2.1.1 (OpenEye Scientific; www.eyesopen.com). The molecule library was docked using FRED from OEDOCKING, version 4.2.1.1 (OpenEye Scientific.; www.eyesopen.com). The structures were drawn using ChemDraw 20.0 (PerkinElmer, https://perkinelmerinformatics.com). NMR spectra were analyzed with MestReNova v12.0.0–20080 (Mestrelab Research; https://mestrelab.com). HPLC-MS spectra were analyzed with Advion Data Express v6.4.10.3 (Advion Interchim Scientific; https://www.advion.com). Patch-clamp data were analyzed using the software package pClamp 10.7 (Molecular Devices; https://www.moleculardevices.com) and GraphPad Prism 8 (GraphPad; https://www.graphpad.com). This software is distributed under license.

## Supporting Information Available

The following files are available free of charge. Predicted binding mode of the guanidine inhibitor 2GBI in its proposed binding pocket in the H_V_1 VSD. Block of H_V_1 channels by **13**. Block of H_V_1 channels by **13** and ClGBI. The IC_50_ values and dose–response curves for the antiproliferative activities of compounds **13**, **23**–**26**, **32**, **42** and ClGBI evaluated on the triple negative breast cancer cell line MDA-MB-231 and on the human monocytic leukemia cell line THP-1. Synthetic procedures and characterization data for compounds **12**, **14**, **17**, **30**, **37**, **42**, **43** and ClGBI. ^1^H NMR, ^13^C NMR spectra and HPLC chromatograms of the representative tested compounds (PDF)
SMILES molecular formula strings (XLSX)

## ABBREVIATIONS

2GBI: 2-guanidinobenzimidazole
CHO: Chinese hamster ovary cells
ClGBI: 5-chloro-2-guanidinobenzimidazole
DRG: dorsal root ganglion neurons
ECS: extracellular (bath) solution
FBS: fetal bovine serum
HBA: hydrogen bond acceptor
HBD: hydrogen bond donor
HIF: H_V_1 inhibitor flexible
hH_V_1: human voltage-gated proton channel 1
ICS: intracellular (pipet) solution
MTS: 3-(4,5-dimethylthiazol-2-yl)-5-(3-carboxymethoxyphenyl)-2-(4-sulfophenyl)-2*H*-tetrazolium
NADPH: nicotinamide adenine dinucleotide phosphate hydrogen
*n*_*H*_: Hill coefficient
NOD/SCID: nonobese diabetic/severe combined immunodeficiency mouse
NOX2: NADPH oxidase 2
ROS: reactive oxygen species
SEM: standard error of the mean
TFA: trifluoroacetic acid
VS: Virtual Screening
VSD: voltage-sensing domain

## References

[ref1] Shen Y.; Luo Y.; Liao P.; Zuo Y.; Jiang R. Role of the Voltage-Gated Proton Channel Hv1 in Nervous Systems. Neurosci Bull. 2023, 39 (7), 1157–1172. 10.1007/s12264-023-01053-6. 37029856 PMC10313628

[ref2] Ramsey I. S.; Moran M. M.; Chong J. A.; Clapham D. E. A voltage-gated proton-selective channel lacking the pore domain. Nature 2006, 440 (7088), 1213–1216. 10.1038/nature04700. 16554753 PMC4084761

[ref3] Thomas R. C.; Meech R. W. Hydrogen ion currents and intracellular pH in depolarized voltage-clamped snail neurones. Nature 1982, 299 (5886), 826–828. 10.1038/299826a0. 7133121

[ref4] Kawanabe A.; Takeshita K.; Takata M.; Fujiwara Y. ATP modulates the activity of the voltage-gated proton channel through direct binding interaction. J. Physiol 2023, 601 (18), 4073–4089. 10.1113/JP284175. 37555355

[ref5] Zhang Q.; Ren Y.; Mo Y.; Guo P.; Liao P.; Luo Y.; Mu J.; Chen Z.; Zhang Y.; Li Y.; Yang L.; Liao D.; Fu J.; Shen J.; Huang W.; Xu X.; Guo Y.; Mei L.; Zuo Y.; Liu J.; Yang H.; Jiang R. Inhibiting Hv1 channel in peripheral sensory neurons attenuates chronic inflammatory pain and opioid side effects. Cell Res. 2022, 32 (5), 461–476. 10.1038/s41422-022-00616-y. 35115667 PMC9061814

[ref6] DeCoursey T. E. Voltage-gated proton channels: molecular biology, physiology, and pathophysiology of the H(V) family. Physiol. Rev. 2013, 93 (2), 599–652. 10.1152/physrev.00011.2012. 23589829 PMC3677779

[ref7] Ribeiro-Silva L.; Queiroz F. O.; da Silva A. M.; Hirata A. E.; Arcisio-Miranda M. Voltage-Gated Proton Channel in Human Glioblastoma Multiforme Cells. ACS Chem. Neurosci. 2016, 7 (7), 864–869. 10.1021/acschemneuro.6b00083. 27225904

[ref8] Asuaje A.; Smaldini P.; Martin P.; Enrique N.; Orlowski A.; Aiello E. A.; Gonzalez Leon C.; Docena G.; Milesi V. The inhibition of voltage-gated H(+) channel (HVCN1) induces acidification of leukemic Jurkat T cells promoting cell death by apoptosis. Pflugers Arch 2017, 469 (2), 251–261. 10.1007/s00424-016-1928-0. 28013412

[ref9] El Chemaly A.; Okochi Y.; Sasaki M.; Arnaudeau S.; Okamura Y.; Demaurex N. VSOP/Hv1 proton channels sustain calcium entry, neutrophil migration, and superoxide production by limiting cell depolarization and acidification. J. Exp Med. 2010, 207 (1), 129–139. 10.1084/jem.20091837. 20026664 PMC2812533

[ref10] Hondares E.; Brown M. A.; Musset B.; Morgan D.; Cherny V. V.; Taubert C.; Bhamrah M. K.; Coe D.; Marelli-Berg F.; Gribben J. G.; Dyer M. J.; DeCoursey T. E.; Capasso M. Enhanced activation of an amino-terminally truncated isoform of the voltage-gated proton channel HVCN1 enriched in malignant B cells. Proc. Natl. Acad. Sci. U. S. A. 2014, 111 (50), 18078–18083. 10.1073/pnas.1411390111. 25425665 PMC4273330

[ref11] Chaves G.; Jardin C.; Derst C.; Musset B. Voltage-Gated Proton Channels in the Tree of Life. Biomolecules 2023, 13 (7), 1035 10.3390/biom13071035. 37509071 PMC10377628

[ref12] Cong S.; Zhang J.; Pan F.; Pan L.; Zhang A.; Ma J. Research progress on ion channels and their molecular regulatory mechanisms in the human sperm flagellum. FASEB J. 2023, 37 (7), e23052 10.1096/fj.202300756R. 37352114

[ref13] Zhao R.; Kennedy K.; De Blas G. A.; Orta G.; Pavarotti M. A.; Arias R. J.; de la Vega-Beltrán J. L.; Li Q.; Dai H.; Perozo E.; Mayorga L. S.; Darszon A.; Goldstein S. A. N. Role of human Hv1 channels in sperm capacitation and white blood cell respiratory burst established by a designed peptide inhibitor. Proc. Natl. Acad. Sci. U. S. A. 2018, 115 (50), e11847–e11856. 10.1073/pnas.1816189115. 30478045 PMC6294887

[ref14] Lazaridis T. Proton Paths in Models of the Hv1 Proton Channel. J. Phys. Chem. B 2023, 7937 10.1021/acs.jpcb.3c03960. 37695850

[ref15] Alvear-Arias J. J.; Pena-Pichicoi A.; Carrillo C.; Fernandez M.; Gonzalez T.; Garate J. A.; Gonzalez C. Role of voltage-gated proton channel (Hv1) in cancer biology. Front. Pharmacol. 2023, 14, 1175702 10.3389/fphar.2023.1175702. 37153807 PMC10157179

[ref16] Tombola F.; Ulbrich M. H.; Isacoff E. Y. The voltage-gated proton channel Hv1 has two pores, each controlled by one voltage sensor. Neuron 2008, 58 (4), 546–556. 10.1016/j.neuron.2008.03.026. 18498736 PMC2430592

[ref17] Geragotelis A. D.; Wood M. L.; Göddeke H.; Hong L.; Webster P. D.; Wong E. K.; Freites J. A.; Tombola F.; Tobias D. J. Voltage-dependent structural models of the human Hv1 proton channel from long-timescale molecular dynamics simulations. Proc. Natl. Acad. Sci. U. S. A. 2020, 117 (24), 13490–13498. 10.1073/pnas.1920943117. 32461356 PMC7306757

[ref18] Chamberlin A.; Qiu F.; Wang Y.; Noskov S. Y.; Peter Larsson H. Mapping the gating and permeation pathways in the voltage-gated proton channel Hv1. J. Mol. Biol. 2015, 427 (1), 131–145. 10.1016/j.jmb.2014.11.018. 25481746 PMC4381436

[ref19] Bagal S. K.; Brown A. D.; Cox P. J.; Omoto K.; Owen R. M.; Pryde D. C.; Sidders B.; Skerratt S. E.; Stevens E. B.; Storer R. I.; Swain N. A. Ion channels as therapeutic targets: a drug discovery perspective. J. Med. Chem. 2013, 56 (3), 593–624. 10.1021/jm3011433. 23121096

[ref20] Pupo A.; Gonzalez León C. In pursuit of an inhibitory drug for the proton channel. Proc. Natl. Acad. Sci. U. S. A. 2014, 111 (27), 9673–9674. 10.1073/pnas.1408808111. 24920591 PMC4103314

[ref21] Boedtkjer E.; Pedersen S. F. The Acidic Tumor Microenvironment as a Driver of Cancer. Annu. Rev. Physiol. 2020, 82, 103–126. 10.1146/annurev-physiol-021119-034627. 31730395

[ref22] Petho Z.; Najder K.; Carvalho T.; McMorrow R.; Todesca L. M.; Rugi M.; Bulk E.; Chan A.; Lowik C.; Reshkin S. J.; Schwab A. pH-Channeling in Cancer: How pH-Dependence of Cation Channels Shapes Cancer Pathophysiology. Cancers 2020, 12 (9), 2484 10.3390/cancers12092484. 32887220 PMC7565548

[ref23] Wang Y.; Li S. J.; Wu X.; Che Y.; Li Q. Clinicopathological and biological significance of human voltage-gated proton channel Hv1 protein overexpression in breast cancer. J. Biol. Chem. 2012, 287 (17), 13877–13888. 10.1074/jbc.M112.345280. 22367212 PMC3340163

[ref24] El Chemaly A.; Jaquet V.; Cambet Y.; Caillon A.; Cherpin O.; Balafa A.; Krause K. H.; Demaurex N. Discovery and validation of new Hv1 proton channel inhibitors with onco-therapeutic potential. Biochimica et biophysica acta. Molecular cell research 2023, 1870 (3), 119415 10.1016/j.bbamcr.2022.119415. 36640925

[ref25] Wang Y.; Wu X.; Li Q.; Zhang S.; Li S. J. Human voltage-gated proton channel hv1: a new potential biomarker for diagnosis and prognosis of colorectal cancer. PLoS One 2013, 8 (8), e70550 10.1371/journal.pone.0070550. 23940591 PMC3734282

[ref26] Bare D. J.; Cherny V. V.; DeCoursey T. E.; Abukhdeir A. M.; Morgan D. Expression and function of voltage gated proton channels (Hv1) in MDA-MB-231 cells. PLoS One 2020, 15 (5), e0227522 10.1371/journal.pone.0227522. 32374759 PMC7202653

[ref27] Wang Y.; Li S. J.; Pan J.; Che Y.; Yin J.; Zhao Q. Specific expression of the human voltage-gated proton channel Hv1 in highly metastatic breast cancer cells, promotes tumor progression and metastasis. Biochem. Biophys. Res. Commun. 2011, 412 (2), 353–359. 10.1016/j.bbrc.2011.07.102. 21821008

[ref28] Chaves G.; Bungert-Plümke S.; Franzen A.; Mahorivska I.; Musset B. Zinc modulation of proton currents in a new voltage-gated proton channel suggests a mechanism of inhibition. Febs j 2020, 287 (22), 4996–5018. 10.1111/febs.15291. 32160407 PMC7754295

[ref29] Takeshita K.; Sakata S.; Yamashita E.; Fujiwara Y.; Kawanabe A.; Kurokawa T.; Okochi Y.; Matsuda M.; Narita H.; Okamura Y.; Nakagawa A. X-ray crystal structure of voltage-gated proton channel. Nature structural & molecular biology 2014, 21 (4), 352–357. 10.1038/nsmb.2783. 24584463

[ref30] Hong L.; Pathak M. M.; Kim I. H.; Ta D.; Tombola F. Voltage-sensing domain of voltage-gated proton channel Hv1 shares mechanism of block with pore domains. Neuron 2013, 77 (2), 274–287. 10.1016/j.neuron.2012.11.013. 23352164 PMC3559007

[ref31] Hong L.; Kim I. H.; Tombola F. Molecular determinants of Hv1 proton channel inhibition by guanidine derivatives. Proc. Natl. Acad. Sci. U. S. A. 2014, 111 (27), 9971–9976. 10.1073/pnas.1324012111. 24912149 PMC4103315

[ref32] Zhao C.; Hong L.; Galpin J. D.; Riahi S.; Lim V. T.; Webster P. D.; Tobias D. J.; Ahern C. A.; Tombola F. HIFs: New arginine mimic inhibitors of the Hv1 channel with improved VSD-ligand interactions. J. Gen. Physiol. 2021, 153 (9), e202012832 10.1085/jgp.202012832. 34228044 PMC8263924

[ref33] Zhao C.; Hong L.; Riahi S.; Lim V. T.; Tobias D. J.; Tombola F. A novel Hv1 inhibitor reveals a new mechanism of inhibition of a voltage-sensing domain. J. Gen. Physiol. 2021, 153 (9), e202012833 10.1085/jgp.202012833. 34228045 PMC8263925

[ref34] Szanto T. G.; Feher A.; Korpos E.; Gyöngyösi A.; Kállai J.; Mészáros B.; Ovari K.; Lányi Á.; Panyi G.; Varga Z. 5-Chloro-2-Guanidinobenzimidazole (ClGBI) Is a Non-Selective Inhibitor of the Human H(V)1 Channel. Pharmaceuticals 2023, 16 (5), 656 10.3390/ph16050656. 37242439 PMC10222378

[ref35] Hawkins P. C.; Skillman A. G.; Warren G. L.; Ellingson B. A.; Stahl M. T. Conformer generation with OMEGA: algorithm and validation using high quality structures from the Protein Databank and Cambridge Structural Database. J. Chem. Inf Model 2010, 50 (4), 572–584. 10.1021/ci100031x. 20235588 PMC2859685

[ref36] McGann M. FRED pose prediction and virtual screening accuracy. J. Chem. Inf Model 2011, 51 (3), 578–596. 10.1021/ci100436p. 21323318

[ref37] McGann M. FRED and HYBRID docking performance on standardized datasets. J. Comput. Aided Mol. Des 2012, 26 (8), 897–906. 10.1007/s10822-012-9584-8. 22669221

[ref38] Hodnik Ž.; Tomašić T.; Mašič L. P.; Chan F.; Kirby R. W.; Madge D. J.; Kikelj D. Novel state-dependent voltage-gated sodium channel modulators, based on marine alkaloids from Agelas sponges. Eur. J. Med. Chem. 2013, 70, 154–164. 10.1016/j.ejmech.2013.07.034. 24148992

[ref39] Hodnik Ž.; Łoś J. M.; Žula A.; Zidar N.; Jakopin Ž.; Łoś M.; Sollner Dolenc M.; Ilaš J.; Węgrzyn G.; Peterlin Mašič L.; Kikelj D. Inhibition of biofilm formation by conformationally constrained indole-based analogues of the marine alkaloid oroidin. Bioorg. Med. Chem. Lett. 2014, 24 (11), 2530–2534. 10.1016/j.bmcl.2014.03.094. 24755428

[ref40] Zidar N.; Jakopin Ž.; Madge D. J.; Chan F.; Tytgat J.; Peigneur S.; Dolenc M. S.; Tomašić T.; Ilaš J.; Mašič L. P.; Kikelj D. Substituted 4-phenyl-2-aminoimidazoles and 4-phenyl-4,5-dihydro-2-aminoimidazoles as voltage-gated sodium channel modulators. Eur. J. Med. Chem. 2014, 74, 23–30. 10.1016/j.ejmech.2013.12.034. 24440379

[ref41] Zidar N.; Montalvão S.; Hodnik Ž.; Nawrot D. A.; Žula A.; Ilaš J.; Kikelj D.; Tammela P.; Mašič L. P. Antimicrobial activity of the marine alkaloids, clathrodin and oroidin, and their synthetic analogues. Mar Drugs 2014, 12 (2), 940–963. 10.3390/md12020940. 24534840 PMC3944524

[ref42] Hamill O. P.; Marty A.; Neher E.; Sakmann B.; Sigworth F. J. Improved patch-clamp techniques for high-resolution current recording from cells and cell-free membrane patches. Pflugers Arch 1981, 391 (2), 85–100. 10.1007/BF00656997. 6270629

[ref43] Peigneur S.; Zula A.; Zidar N.; Chan-Porter F.; Kirby R.; Madge D.; Ilaš J.; Kikelj D.; Tytgat J. Action of clathrodin and analogues on voltage-gated sodium channels. Mar Drugs 2014, 12 (4), 2132–2143. 10.3390/md12042132. 24714127 PMC4012458

[ref44] Tomašič T.; Nabergoj D.; Vrbek S.; Zidar N.; Jakopin Ž.; Žula A.; Hodnik Ž.; Jukič M.; Anderluh M.; Ilaš J.; Dolenc M. S.; Peluso J.; Ubeaud-Séquier G.; Muller C. D.; Mašič L. P.; Kikelj D. Analogues of the marine alkaloids oroidin, clathrodin, and hymenidin induce apoptosis in human HepG2 and THP-1 cancer cells. MedChemComm 2015, 6 (1), 105–110. 10.1039/C4MD00286E.

[ref45] DeCoursey T. E.; Cherny V. V. Voltage-activated proton currents in human THP-1 monocytes. J. Membr. Biol. 1996, 152 (2), 131–140. 10.1007/s002329900092. 9139124

