# Correction to “Identification of a Novel Structural Class of HV1 Inhibitors by Structure-Based Virtual Screening”

**DOI:** 10.1021/acs.jcim.4c02211

**Published:** 2024-12-10

**Authors:** Martina Piga, Zoltan Varga, Adam Feher, Ferenc Papp, Eva Korpos, Kavya C. Bangera, Rok Frlan, Janez Ilaš, Jaka Dernovšek, Tihomir Tomašič, Nace Zidar

**Affiliations:** † Faculty of Pharmacy, University of Ljubljana, Aškerčeva cesta 7, Ljubljana 1000, Slovenia; ‡ Faculty of Medicine, Department of Biophysics and Cell Biology, University of Debrecen, Egyetem tér 1, Debrecen H-4032, Hungary; § HUN-REN−UD Cell Biology and Signaling Research Group, Egyetem tér 1, Debrecen H-4032, Hungary

The department has been added to the University of Debrecen affiliation.

