# Supplementary material for: Identification of a Novel Structural Class of HV1 Inhibitors by Structure-Based Virtual Screening
Source: J Chem Inf Model. 2024 Jun 8;64(12):4850–62. doi: 10.1021/acs.jcim.4c00240 (PMC11200261; doi:10.1021/acs.jcim.4c00240)
Supplement: Supplementary file 1 — ci4c00240_si_001.pdf [file ci4c00240_si_001.pdf]

SUPPORTING INFORMATION

Identification of a Novel Structural Class of H_V1 Inhibitors by Structure-Based Virtual Screening

*Martina Piga[†], Zoltan Varga[‡], Adam Feher[‡], Ferenc Papp[‡], Eva Korpos[‡§], Kavya C. Bangera[‡], Rok Frlan[†], Janez Ilaš[†], Jaka Dernovšek[†], Tihomir Tomašič[†], Nace Zidar[†]**

[†]University of Ljubljana, Faculty of Pharmacy, Aškerčeva cesta 7, 1000 Ljubljana, Slovenia

[‡]University of Debrecen, Faculty of Medicine, Egyetem tér 1. H-4032 Debrecen, Hungary

[§]HUN-REN–UD Cell Biology and Signaling Research Group, Egyetem tér 1. H-4032 Debrecen, Hungary

* Corresponding author: University of Ljubljana, Faculty of Pharmacy, 1000 Ljubljana, Slovenia.
E-mail address: nace.zidar@ffa.uni-lj.si.

Table of Contents:

1. Predicted binding mode of 2GBI
2. Block of H_V1 channels by **13**
3. Block of H_V1 channels by **13** and ClGBI
4. Changes in the tail currents
5. Inhibitory effect of **44** on H_V1 channels
6. Antiproliferative activities on the MDA-MB-231 breast cancer cell line
7. Dose-response curves on the MDA-MB-231 breast cancer cell line
8. Antiproliferative activities on the human monocytic leukemia cell line THP-1
9. Dose-response curves on the human monocytic leukemia cell line THP-1
10. Chemistry - synthetic procedures and characterization data
11. ^{1}H NMR, ^{13}C NMR spectra and HPLC chromatograms of the representative tested compounds
12. SMILES for all tested compounds
13. References

1. Predicted binding mode of 2GBI

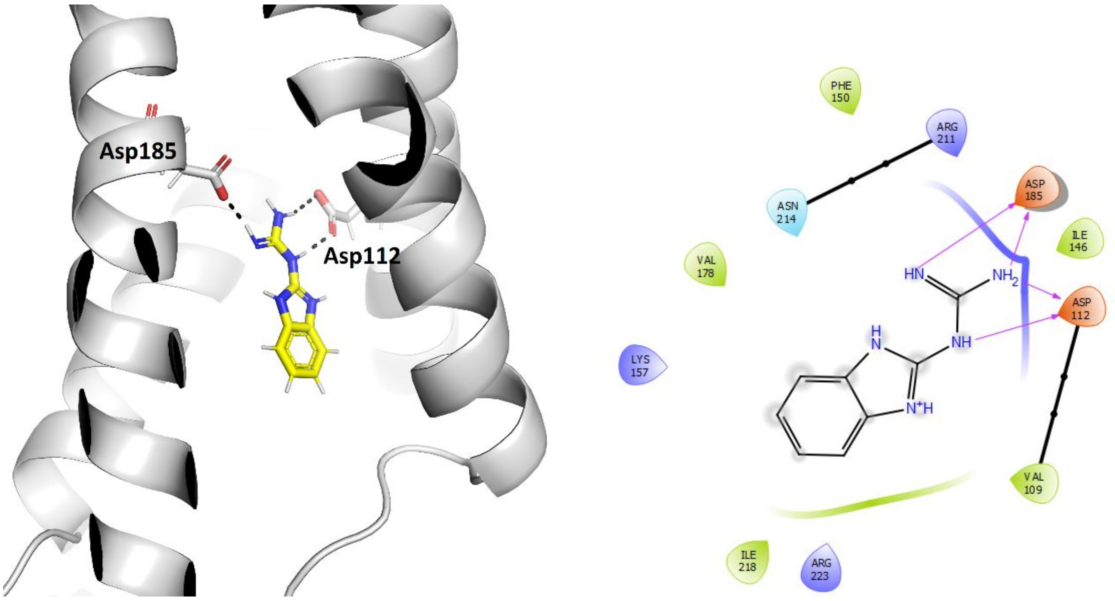

Figure S1. Predicted binding mode of the guanidine inhibitor 2GBI in its proposed binding pocket in the H_V1 VSD, predicted by docking with FRED.[1] The ligand and the neighboring protein side chains are shown as stick models. Hydrogen bonds are represented by dashed black lines and magenta arrows. For clarity, only key amino acids are shown.

2. Block of H_V1 channels by 13

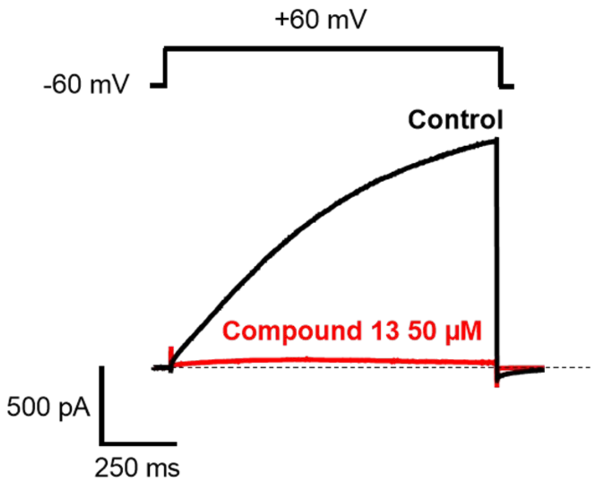

Figure S2. Block of H_V1 channels by **13**. Whole-cell currents were evoked by a 1-second depolarizing step to +60 mV. The traces show H^+ current in the absence (black) and presence of 50 µM **13** (red) at equilibrium block.

3. Block of H_V1 channels by 13 and ClGBI

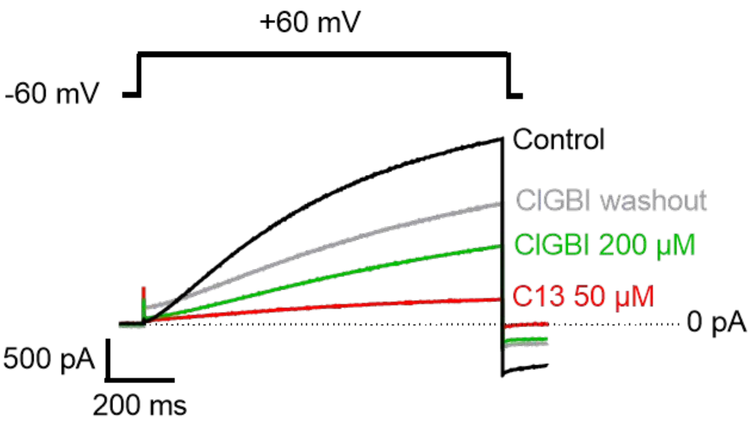

Figure S3. Block of H_V1 channels by **13** and ClGBI. Whole-cell currents were evoked by a 1-second depolarizing step to +60 mV. The traces show H^+ current in the absence (black) and presence of 50 µM **13** (red) at equilibrium block, and of 200 µM ClGBI (green) used as a positive control.

4. Changes in the tail currents

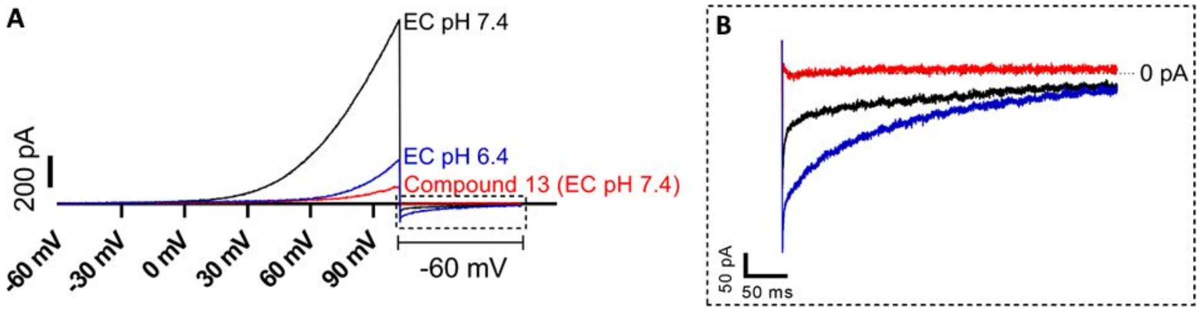

Figure S4. The changes in the tail currents were determined by a voltage step to -60 mV after the ramp protocol (A and B). Panel B shows the magnified tail current from panel A. Under control conditions (black trace), the ΔpH (= EC pH – IC pH) was 1 and the calculated equilibrium potential for H^+ was -58 mV. Changing the perfusion to pH 6.4 extracellular solution (blue trace, ΔpH=0) shifts the opening voltage to more positive membrane potentials, decreases the peak current at +100 mV and increases the tail current. When compound **13** (red trace, diluted in control solution) was applied, both the peak current at +100 mV and the tail current decreased.

5. Inhibitory effect of 44 on H_V1 channels

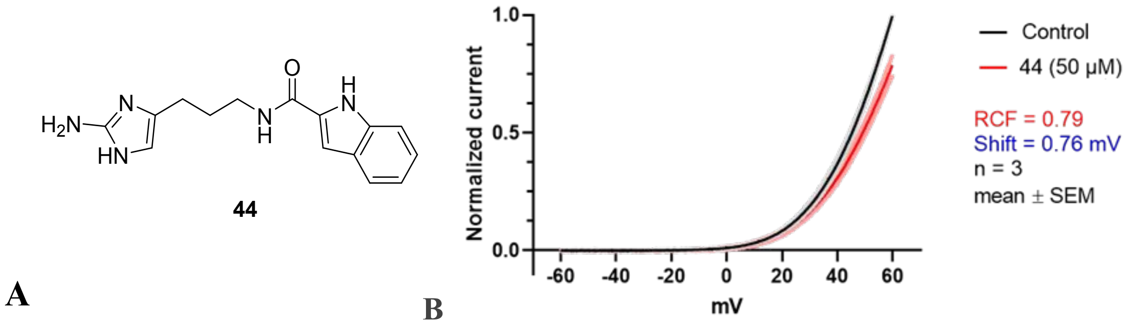

Figure S5. The structure of compound **44**, an analogue of **25**, in which the central phenyl ring is replaced by an alkyl chain (A). Inhibitory effect of **44** on H_V1 channels (B). The normalized current is shown on the Y-axis and the applied voltage on the X-axis. The control

is the black curve with grey error bars, while **44** is the red curve with pink error bars (n=3, mean ± SEM). For each measurement, all current samples were normalized to the current amplitude under control conditions.

6. Antiproliferative activities on the MDA-MB-231 breast cancer cell line

Table S1. IC_{50} values for the antiproliferative activities of compounds **13**, **23-26**, **32**, **42** and **ClGBI** evaluated on the triple negative MDA-MB-231 breast cancer cell line. 17-DMAG, a known Hsp90 inhibitor, was used as a positive control. Data represent mean ± SD of at least two independent experiments performed in triplicate.

ID	IC_{50} (µM)	ID	IC_{50} (µM)
**13**	9.0 ± 1.0	**32**	31.9 ± 2.2
**23**	22.1 ± 1.8	**42**	29.0 ± 1.7
**24**	17.1 ± 0.3	**ClGBI**	>50
**25**	23.0 ± 1.3	**17-DMAG**	0.9 ± 0.1
**26**	>50		

7. Dose-response curves on the MDA-MB-231 breast cancer cell line

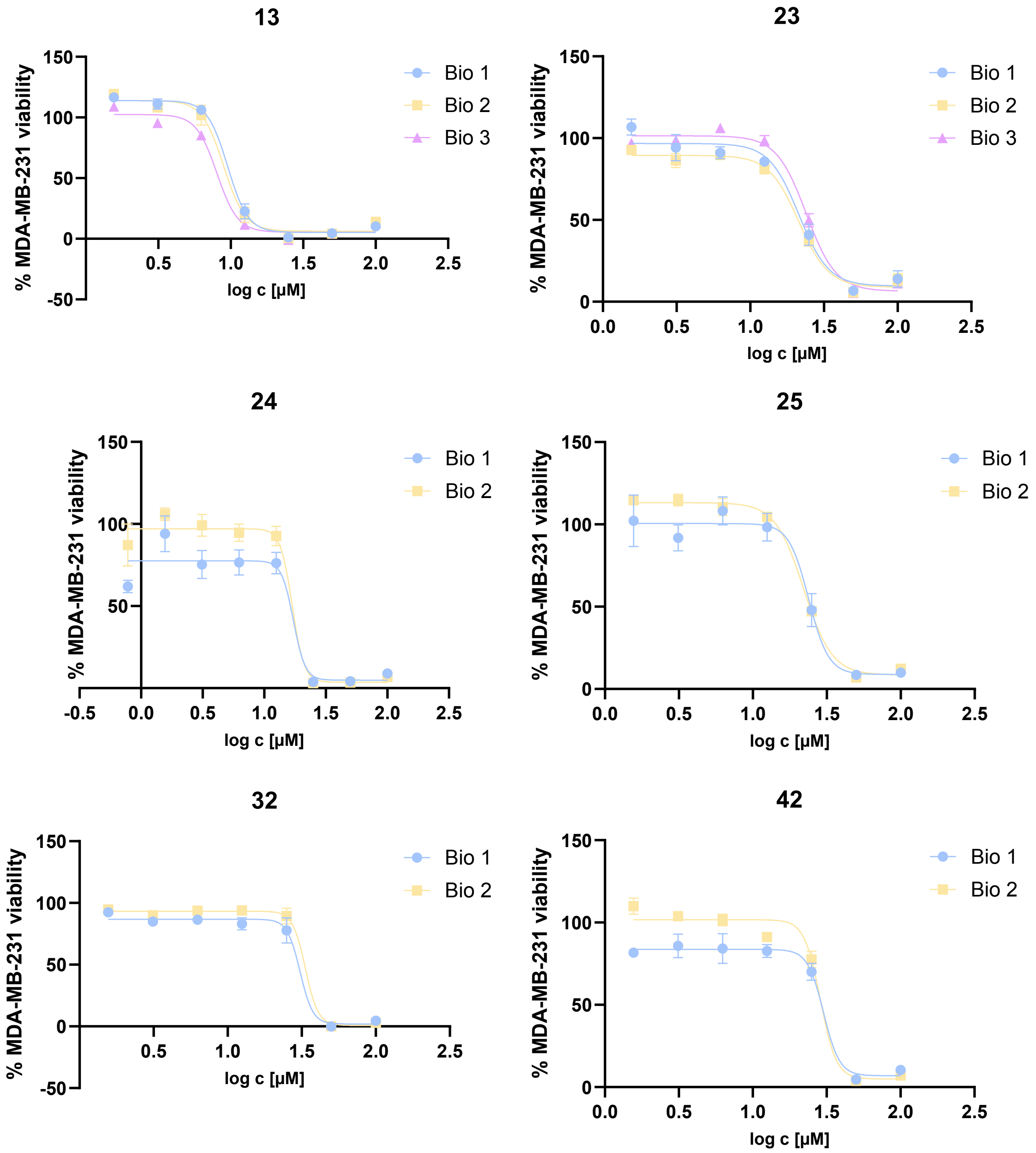

Figure S6. Dose-response curves for compounds **13**, **23-25**, **32** and **42** in MTS assay on the triple negative MDA-MB-231 breast cancer cell line, shown for at least two independent experiments, each performed in triplicate.

8. Antiproliferative activities on the human monocytic leukemia cell line THP-1

Table S2. IC_{50} values for the antiproliferative activities of compounds **13**, **23-26**, **32**, **42** and **ClGBI** evaluated on the human monocytic leukemia cell line THP-1. PU-H71, a known Hsp90 inhibitor, was used as a positive control. Data represent mean ± SD of at least two independent experiments performed in triplicate.

ID	IC_{50} (μM)	ID	IC_{50} (μM)
**13**	8.1 ± 4.3	**32**	35.0 ± 9.4
**23**	16.2 ± 3.3	**42**	31.6 ± 6.5
**24**	15.8 ± 1.6	**ClGBI**	>50
**25**	17.6 ± 1.4	**PU-H71**	0.27 ± 0.05
**26**	>50		

9. Dose-response curves on the human monocytic leukemia cell line THP-1

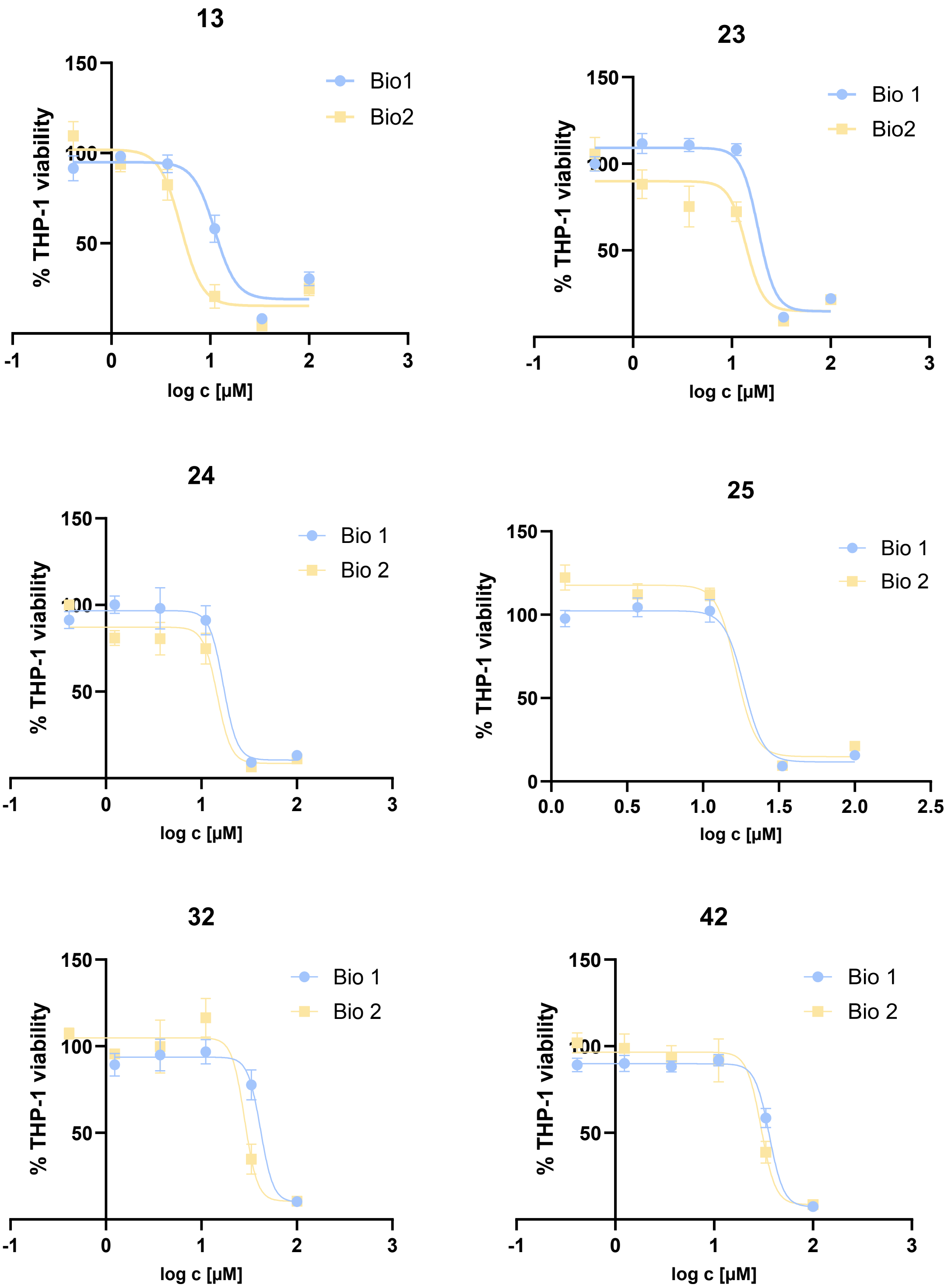

Figure S7. Dose-response curves for compounds **13**, **23-25**, **32** and **42** in MTS assay on the human monocytic leukemia cell line THP-1, shown for at least two independent experiments, each performed in triplicate.

10. Chemistry - synthetic procedures and characterization data

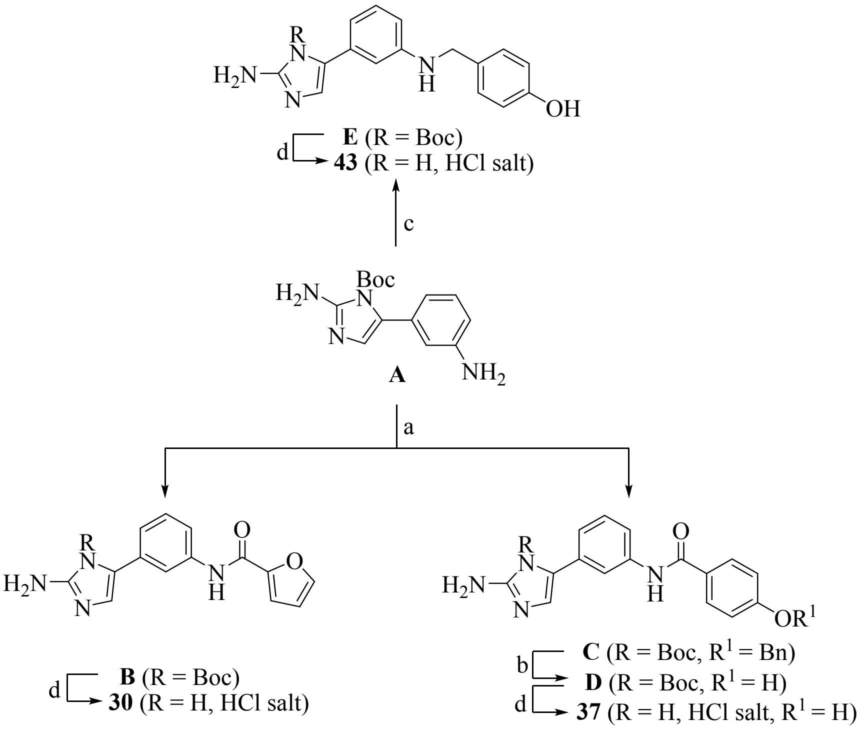

Scheme 1. Reagents and conditions: (a) corresponding carboxylic acid, TBTU, NMM, CH_2Cl_2, 35 °C, 24 h; (b) H_2/Pd-C, THF/MeOH, rt, 5 h; (c) 4-hydroxybenzaldehyde, $NaBH(OAc)_3$, CH_3COOH, CH_2Cl_2, rt, 15 h; (d) $HCl_{(g)}$, THF/EtOH, rt, 5 h. For details on the synthesis of compound **A** please refer to N. Zidar, Ž. Jakopin, D. J. Madge, F. Chan, J. Tytgat, S. Peigneur, M. S. Dolenc, T. Tomašić, J. Ilaš, L. P. Mašič, D. Kikelj, Eur. J. Med. Chem. 2014, 74, 23-30.

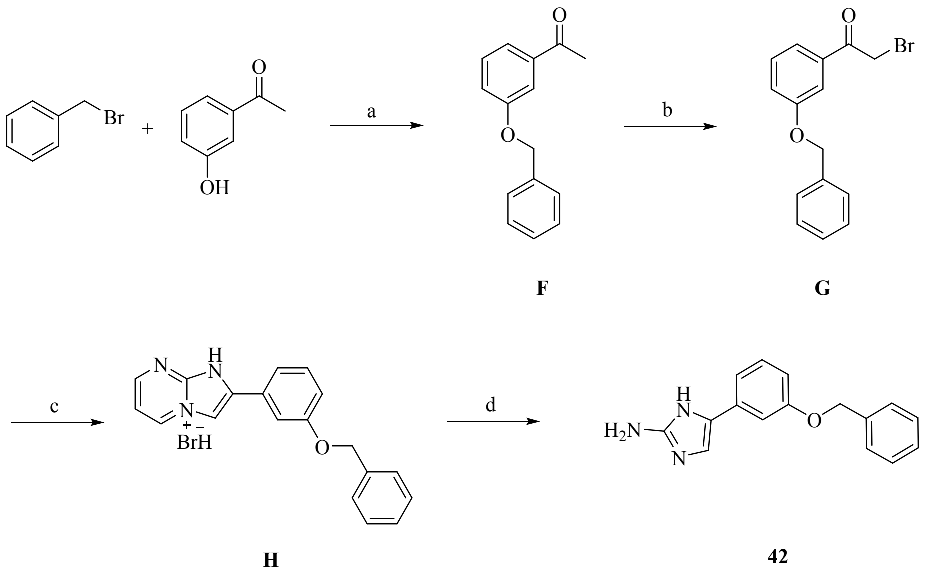

Scheme 2. Reagents and conditions: (a) K_2CO_3, CH_3CN, 70 °C, 2 h; (b) Br_2, anhydrous MeOH, rt, 15 h; (c) 2-aminopyrimidine, DMAP, CH_3CN, 85 °C, 15 h; (d) hydrazine hydrate, abs. EtOH, MW: 120 °C, 80 min.

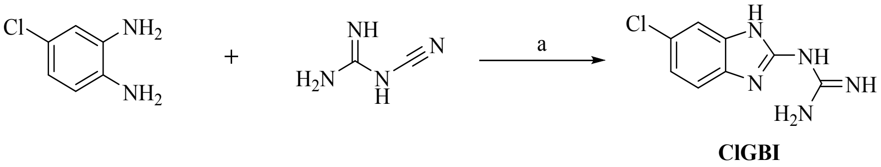

Scheme 3. Reagents and conditions: (a) conc. HCl, H_2O, 100 ˚C, 16 h.

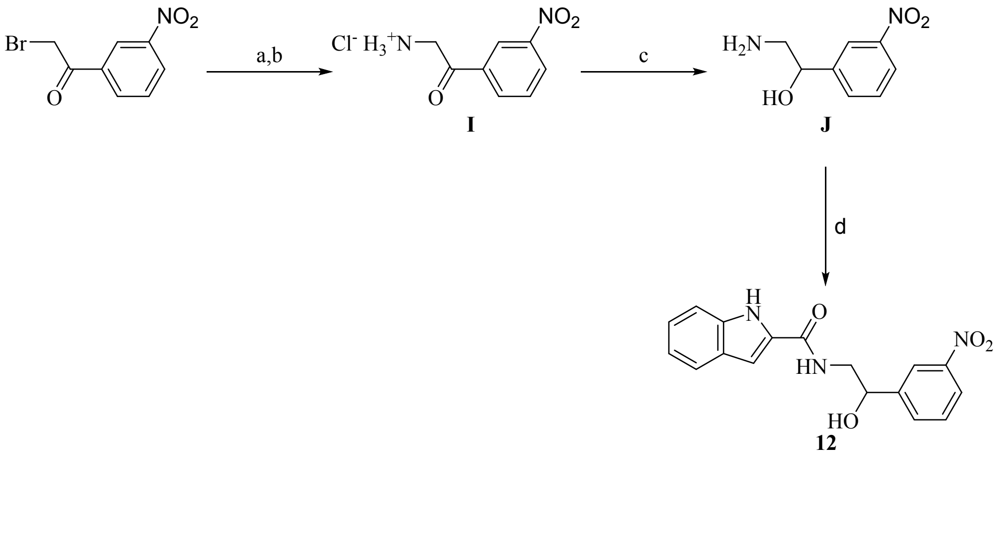

Scheme 4. Reagents and conditions: (a) HMTA, CH_2Cl_2, rt, 3 h; (b) conc. HCl, EtOH, rt, 2 d; (c) $NaBH_4$, MeOH, rt, 12 h; (d) indole-2-carboxylic acid, EDC, HOBt, NMM, DMF, rt, 24 h.

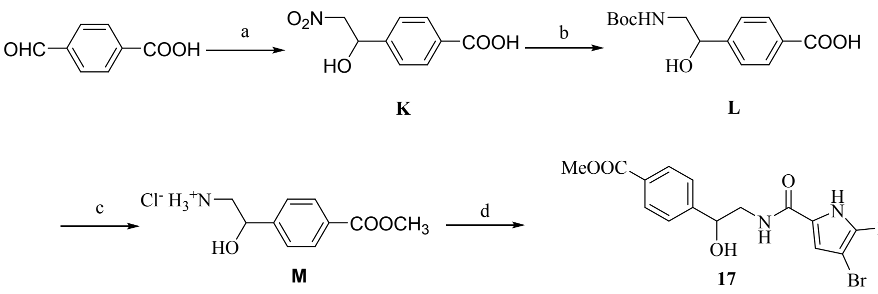

Scheme 5. Reagents and conditions: (a) CH_3NO_2, LiOH, THF, H_2O, 0 °C, 3 d; (b) H_2, Pd/C, Boc_2O, MeOH, 3.5 h; (c) $SOCl_2$, MeOH, 0 °C to rt, 12 h; (d) indole-2-carboxylic acid, EDC, HOBt, NMM, DMF, rt, 24 h.

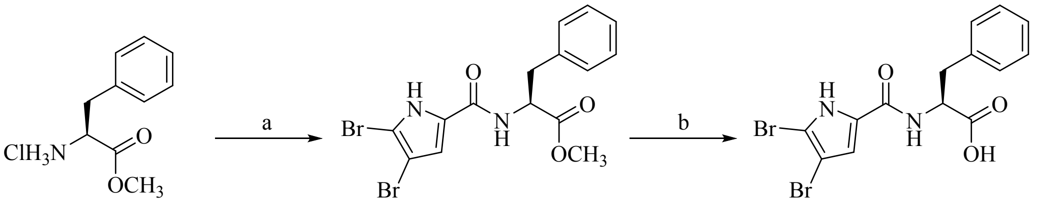

Scheme 6. Reagents and conditions: (a) 2,2,2-trichloro-1-(4,5-dibromo-1*H*-pyrrol-2-yl)ethan-1-one, rt, CH_3CN, 3 d; (b) 1,4-dioxane, 1 M LiOH, rt, 24 h.

10.1. General procedure for the syntheses of compounds B and C (with B as an example). To a suspension of furan-2-carboxylic acid (147 mg, 1.31 mmol) and 2-(1*H*-Benzotriazole-1-yl)-1,1,3,3-tetramethyluronium tetrafluoroborate (456 mg, 1.42 mmol) in dichloromethane (5 mL), *N*-methylmorpholine (601 μL, 5.47 mmol) was added, and the mixture stirred at rt for 0.5

h upon which a clear solution was formed. Compound **A** (300 mg, 1.09 mmol) was added and the mixture stirred at 35 ˚C overnight. The solvent was evaporated *in vacuo*, the residue dissolved in ethyl acetate (30 mL), and washed successively with water (2 × 10 mL), saturated aqueous $NaHCO_3$ solution (2 × 10 mL), and brine (1 × 10 mL). The organic phase was dried over Na_2SO_4, filtered and the solvent evaporated under reduced pressure. The crude product was purified by flash column chromatography using ethyl acetate/petroleum ether and then dichloromethane/methanol as eluents, to afford **B**.

10.1.1. *tert*-Butyl 2-amino-5-(3-(furan-2-carboxamido)phenyl)-1*H*-imidazole-1-carboxylate (B). Yield, 38% (0.153 g); brown solid; ^1H NMR (DMSO-d_6) δ 1.59 (s, 9H, *t*-Bu), 6.63 (s, 2H, NH_2), 6.71–6.72 (m, 1H, Fur-H), 7.27 (s, 1H, Ar-H), 7.31 (t, J = 7.9 Hz, 1H, Ar-H), 7.37 (dd, J = 3.5 Hz, J = 0.8 Hz, 1H, Fur-H), 7.46–7.48 (m, 1H, Ar-H), 7.63–7.65 (m, 1H, Ar-H), 7.95 (dd, J = 1.7, 0.8 Hz, 1H, Fur-H), 8.11 (t, J = 1.9 Hz, 1H, Ar-H), 10.18 (s, 1H, NH). MS (ESI+) m/z (%) = 268.6 ([MH-*t*-Bu]$^+$).

10.1.2. *tert*-Butyl 2-amino-5-(3-(4-(benzyloxy)benzamido)phenyl)-1*H*-imidazole-1-carboxylate (C). Yield, 14% (0.189 g); white solid; ^1H NMR (DMSO-d_6) δ 1.60 (s, 9H, *t*-Bu), 5.22 (s, 2H, CH_2), 6.62 (s, 2H, NH_2), 7.12–7.18 (m, 2H, Ar-H), 7.26 (s, 1H, Ar-H), 7.31 (t, J = 7.9 Hz, 1H, Ar-H), 7.34–7.38 (m, 1H, Ar-H), 7.39–7.51 (m, 5H, Ar-H), 7.64–7.67 (m, 1H, Ar-H), 7.94-9.02 (m, 2H, Ar-H), 8.14 (t, J = 1.9 Hz, 1H, Ar-H), 10.10 (s, 1H, NH). MS (ESI+) m/z (%) = 384.5 ([MH-*t*-Bu]$^+$).

10.2. Synthesis of compound D. Compound **C** (0.135 g, 0.28 mmol) was dissolved in THF (10 mL) and MeOH (10 mL), Pd/C (95 mg) was added and the reaction mixture was stirred under hydrogen atmosphere for 5 h. The catalyst was filtered off and the solvent removed under reduced pressure to give **D**.

10.2.1. *tert*-Butyl 2-amino-5-(3-(4-hydroxybenzamido)phenyl)-1*H*-imidazole-1-carboxylate (D). Yield, 70% (77 mg); brown solid; ^{1}H NMR (DMSO-d_6) δ 1.60 (s, 9H, *t*-Bu), 6.62 (s, 2H, NH_2), 6.82–6.91 (m, 2H, Ar-H), 7.26 (s, 1H, Ar-H), 7.30 (t, J = 7.9 Hz, 1H, Ar-H), 7.43–7.46 (m, 1H, Ar-H), 7.63–7.66 (m, 1H, Ar-H), 7.86–7.90 (m, 2H, Ar-H), 8.13 (t, J = 1.9 Hz, 1H, Ar-H), 10.00 (s, 1H, OH), 10.11 (s, 1H, NH). MS (ESI+) m/z (%) = 294.6 ([MH-*t*-Bu]$^+$).

10.3. Synthesis of compound E. To a suspension of compound **A** (250 mg, 0.91 mmol) in dichloromethane (15 mL) 4-hydroxybenzaldehyde (145 mg, 1.18 mmol) and glacial acetic acid (52 µL, 0.91 mmol) were added, upon which the mixture became clear. $NaBH(OAc)_3$ (309 mg, 1.46 mmol) was added and the mixture stirred at rt for 15 h. Red opalescent solution was diluted with dichloromethane (20 mL) and washed successively with water (2 × 20 mL), saturated aqueous $NaHCO_3$ solution (2 × 20 mL) and brine (2 × 15 mL). The organic phase was dried over Na_2SO_4, filtered and concentrated *in vacuo*. The crude product was purified by flash column chromatography using ethyl acetate/petroleum ether as an eluent, to afford **E.**

10.3.1. *tert*-Butyl 2-amino-5-(3-((4-hydroxybenzyl)amino)phenyl)-1*H*-imidazole-1-carboxylate (E). Yield, 76% (0.263 g); grey solid; ^{1}H NMR (DMSO-d_6) δ 1.58 (s, 9H, *t*-Bu), 4.15 (d, J = 5.7 Hz, 2H, CH_2), 6.04 (t, J = 5.7 Hz, 1H, NH), 6.44-6.46 (m, 1H, Ar-H), 6.55 (s, 2H, NH_2), 6.69-6.72 (m, 2H, Ar-H), 6.86-6.91 (m, 1H, Ar-H), 6.95-7.03 (m, 2H, Ar-H), 7.10-7.19 (m, 3H, Ar-H), 9.25 (s, 1H, NH), signal for the OH proton not seen. MS (ESI+) m/z (%) = 280.7 ([MH-*t*-Bu]$^+$).

10.4. General procedure for the syntheses of compounds 30, 37 and 43 (with 30 as an example). Solution of compound **B** (84 mg, 0.23 mmol) in a 1:1 mixture of THF and EtOH (15 mL) was saturated with gaseous HCl and stirred at rt for 5 h. The solvent was removed under

reduced pressure, the solid was filtered off and washed with diethyl ether (3 × 5 mL), to afford **30**.

10.4.1. 2-Amino-5-(3-(furan-2-carboxamido)phenyl)-1*H*-imidazol-3-ium chloride (30). Yield, 96% (67 mg); white solid; ^{1}H NMR (DMSO-d_6) δ 6.72–6.73 (m, 1H, Fur-H), 7.31 (s, 1H, Ar-H), 7.40–7.48 (m, 5H, Ar-H, NH_2), 7.60–7.63 (m, 1H, Ar-H), 7.96-7.97 (m, 1H, Fur-H), 8.05-8.06 (m, 1H, Ar-H), 10.36 (s, 1H, NH), 12.15 (s, 1H, NH), 12.83 (s, 1H, NH); ^{13}C NMR (DMSO-d_6) δ 109.97, 112.67, 115.50, 117.10, 120.42, 120.97, 126.79, 128.60, 129.78, 139.49, 146.42, 147.80, 148.34, 156.81. MS (ESI+) *m/z* (%) = 268.5 ([MH-Cl]$^+$). HRMS for $C_{14}H_{12}N_4O_2$: calculated 269.1039; found 269.1031. HPLC: t_r = 4.550 min (97% at 254 nm, 97% at 280 nm).

10.4.2. 2-Amino-5-(3-(4-hydroxybenzamido)phenyl)-1*H*-imidazol-3-ium chloride (37). Yield, 66% (30 mg); white solid; ^{1}H NMR (DMSO-d_6) δ 6.88–6.91 (m, 2H, Ar-H), 7.31 (s, 1H, Ar-H), 7.35–7.50 (m, 4H, Ar-H, NH_2), 7.58–7.60 (m, 1H, Ar-H), 7.84–7.94 (m, 2H, Ar-H), 8.10-8.11 (m, 1H, Ar-H), 10.15 (s, 1H, NH), 10.23 (s, 1H, OH), 12.15 (s, 1H, NH), 12.81 (s, 1H, NH); ^{13}C NMR (DMSO-d_6) δ 108.80, 114.38, 115.94, 118.94, 119.82, 124.38, 125.87, 127.41, 128.58, 129.14, 139.36, 147.22, 160.18, 164.59. MS (ESI+) *m/z* (%) = 294.7 ([MH-Cl]$^+$). HRMS for $C_{16}H_{14}N_4O_2$: calculated 295.11895; found 295.11829. HPLC: t_r = 4.157 min (98% at 254 nm, 97% at 280 nm).

10.4.3. 2-Amino-5-(3-((4-hydroxybenzyl)ammonio)phenyl)-1*H*-imidazol-3-ium chloride (43). Yield, 99% (56 mg); white solid; ^{1}H NMR (DMSO-d_6) δ 4.24-4.27 (m, 2H, CH_2), 6.70-6.74 (m, 2H, Ar-H), 6.75-6.80 (m, 1H, Ar-H), 7.00-7.15 (m, 2H, Ar-H), 7.18-7.24 (m, 3H, Ar-H), 7.28-7.30 (m, 1H, Ar-H), 7.40-7.46 (m, 2H, NH_2), 8.30-10.30 (m, 2H, NH_2), 12.11 (s, 1H,

NH), 12.84 (s, 1H, NH), signal for the OH proton not seen; ^{13}C NMR (DMSO-d_6) δ 49.93, 110.19, 115.57, 118.30, 126.72, 129.23, 130.18, 130.78, 148.23, 157.60. MS (ESI+) *m/z* (%) = 280.7 ([MH-Cl]$^+$). HRMS for $C_{16}H_{16}N_4O$: calculated 281.13969; found 281.13908. HPLC: t_r = 3.470 min (100% at 254 nm, 99% at 280 nm).

10.5. Synthesis of compound F. To a solution of 3-hydroxyacetophenone (0.500 g, 3.67 mmol) in acetonitrile (15 mL), K_2CO_3 (1.015 g, 7.34 mmol) and benzylbromide (458 μL, 3.86 mmol) were added. The reaction mixture was stirred at 70 °C for 2 h. Then the solvent was evaporated *in vacuo*, the residue dissolved in ethyl acetate (25 mL), and washed successively with saturated aqueous $NaHCO_3$ solution (2 × 25 mL), water (2 × 25 mL), and brine (2 × 25 mL). The organic phase was dried over Na_2SO_4, filtered and the solvent evaporated under reduced pressure to afford **F**.

10.5.1. 1-(3-(Benzyloxy)phenyl)ethan-1-one (F). Yield, 99% (0.823 g); off-white solid; ^{1}H NMR ($CDCl_3$) δ 2.62 (s, 3H, CH_3), 5.14 (s, 2H, CH_2), 7.19-7.22 (m, 1H, Ar-H), 7.34-7.50 (m, 6H, Ar-H), 7.55-7.63 (m, 2H, Ar-H). MS (ESI+) *m/z* (%) = 267.6 ([MH-CH_3CN]$^+$).

10.6 Synthesis of compound G. To a solution of **F** (0.750 g, 3.31 mmol) in anhydrous MeOH (10 mL), a solution of Br_2 (221 μL, 4.31 mmol) in anhydrous MeOH (5 mL) was added dropwise. The reaction mixture was stirred at rt for 5 h under argon atmosphere. Then the solvent was evaporated in vacuo and 1 M HCl aq. solution (10 mL) was added. The product was extracted with ethyl acetate (30 mL), and washed with 1 M HCl aq. solution (2 × 10 mL). The organic phase was then washed brine (2 × 15 mL), dried over Na_2SO_4 and filtered. The solvent was evaporated under reduced pressure and the crude product was purified by flash column chromatography using dichloromethane/petroleum ether as an eluent, to afford **G**.

10.6.1. 1-(3-(Benzyloxy)phenyl)-2-bromoethan-1-one (G). Yield, 48% (0.475 g); yellow oil; ^{1}H NMR ($CDCl_3$-*d*) δ 5.15 (s, 2H, CH_2), 4.46 (s, 2H, CH_2), 7.24-7.27 (m, 1H, Ar-H), 7.35-7.49 (m, 6H, Ar-H), 7.58-7.63 (m, 2H, Ar-H). MS (ESI-) *m*/*z* (%) = 303.0 ($[M-H]^-$).

10.7. Synthesis of compound H. To a solution of compound **G** (0.398 g, 1.30 mmol) in acetonitrile (5 mL) 2-aminopyrimidine (0.124 g, 1.30 mmol) and 4-dimethylaminopyridine (1.59 mg, 0.01 mmol) were added. After being stirred at 65 °C for 24 h, the reaction mixture was filtered and washed with acetonitrile (10 mL), to afford **H**.

10.7.1. 2-(3-(Benzyloxy)phenyl)-1*H*-imidazo[1,2-a]pyrimidin-4-ium bromide (H). Yield, 42% (0.211 g); white solid; ^{1}H NMR (DMSO-d_6) δ 5.13 (s, 2H, CH_2), 7.26-7.29 (m, 2H, Ar-H), 7.32–7.52 (m, 7H, Ar-H), 7.67–7.74 (m, 1H, Ar-H), 8.68-8.70 (m, 1H, Ar-H), 8.86-8.89 (m, 1H, Ar-H), 9.19-9.24 (m, 1H, Ar-H). MS (ESI+) *m*/*z* (%) = 302.7 ($[MH-Br]^+$).

10.8. Synthesis of compound 42. To a suspension of **H** (0.172 g, 0.45 mmol) in abs. ethanol (2 mL) in a 10 mL glass vessel, hydrazine hydrate (282 μL, 35% hydrazine in solution, 3.15 mmol) was added, the vessel was sealed, placed in a microwave reactor, and heated at 120 °C for 60 min. The mixture was cooled to rt and the solvent evaporated under reduced pressure. The crude residue was dissolved in ethyl acetate (20 mL), and washed successively with saturated aqueous $NaHCO_3$ solution (2 × 10 mL), and brine (2 × 10 mL). The organic phase was dried over Na_2SO_4 and filtered and residue was purified by flash column chromatography using dichloromethane/methanol/$NH_{3(g)}$ as an eluent, to afford **42**.

10.8.1. 5-(3-(Benzyloxy)phenyl)-1*H*-imidazol-2-amine (42). Yield, 71% (85 mg); off-white solid; ^{1}H NMR (DMSO-d_6) δ 5.11 (s, 2H, CH_2), 5.39 (s, 2H, NH_2), 6.74-6.76 (m, 1H, Ar-H), 6.99 (s, 1H, Ar-H), 7.18-7.19 (m, 2H, Ar-H), 7.25-7.26 (m, 1H, Ar-H), 7.33-7.35 (m, 1H, Ar-

H), 7.38-7.42 (m, 2H, Ar-H), 7.46-7.48 (m, 2H, Ar-H); ^{13}C NMR (DMSO-d_6) δ 69.45, 109.97, 112.00, 116.57, 127.79, 128.12, 128.17, 128.22, 128.22, 128.89, 129.77, 137.79, 150.79, 159.05. MS (ESI+) *m*/*z* (%) = 265.6 ([M+H]$^+$). HRMS for $C_{16}H_{15}N_3O$: calculated 266.12879; found 266.12805. HPLC: t_r = 6.250 min (98% at 254 nm, 98% at 280 nm).

10.9. Synthesis of ClGBI. A mixture of 4-chloro-1,2-phenylendiamine (0. 254 g, 1,78 mmol), dicyandiamide (0.300 g, 3.57 mmol), concentrated hydrochloric acid (12 M, 0.5 mL) and water (2 mL) was heated under reflux for 15 h. The reaction mixture was then treated with 10M NaOH and after 0.5 h cooled down. The suspension was filtered and the obtained water solution was put on ice. When a suspension was formed, it was filtered and the solid was washed with water and dried to afford **ClGBI**.

10.9.1. 1-(6-Chloro-1H-benzo[d]imidazol-2-yl)guanidine (ClGBI). Yield, 30% (0.112 g); yellow solid; ^{1}H NMR (DMSO-d_6) δ 6.90-7.16 (m, 7H, Ar-H, NH, NH_2), 11.12 (s, 1H, NH); ^{13}C NMR (DMSO-d_6) δ 110.21, 114.70, 115.71, 119.44, 119.52, 124.13, 159.46, 160.67. MS (ESI+) m/z (%) = 209.8 ([M+H]+). HRMS for $C_8H_8ClN_5$: calculated 210.05410; found 210.05429. HPLC: t_r = 4.573 min (99% at 254 nm, 99% at 280 nm).

10.10. Synthesis of compound I. Compound **I** was prepared using a modified literature procedure.[2] To a solution of 2-bromo-1-(4-nitrophenyl)ethanone (10.8 g, 44.3 mmol, 1.0 equiv) in dichloromethane (100 mL), hexamethylenetetramine (8.71 g, 62.1 mmol, 1.4 equiv) was added and stirred at room temperature for 3 hours. After filtration of the suspension, concentrated hydrochloric acid (17 mL) and ethanol (70 mL) were added, and the mixture was allowed to stand for 2 days at room temperature. The resulting suspension was then filtered

and washed with water to obtain the crude product, which was subsequently recrystallized from water.

10.10.1. 2-Amino-1-(3-nitrophenyl)ethan-1-one hydrochloride (**I**). Yield, 56%; yellow crystals; Rf (CH_2Cl_2:MeOH:Et_3N = 7:1:0.1): 0.28; ^{1}H NMR (DMSO-d_6): δ 4.69 (s, 2H, CH_2), 8.25 (d, J = 8.9 Hz, 2H, ArH), 8.35 (d, J = 8.9 Hz, 1H, ArH), 8.67 (s, 3H, NH_3) ppm. ^{13}C NMR (DMSO-d_6): δ 45.4, 124.1, 129.9, 138.3, 150.6, 192.4 ppm.

10.11. Synthesis of compound J. Compound **J** was prepared using a modified literature procedure.[3] To a solution of compound **I** (5.0 g, 23.1 mmol, 1.0 equiv.) in methanol (250 mL), $NaBH_4$ (2.62 g, 69.3 mmol, 3 equiv.) was added at 0°C. The temperature was raised to room temperature and stirred for 12 hours. A 10% NH_4Cl solution (10 mL) was added and the mixture was evaporated to dryness. The crude solid was dissolved in 1 M NaOH (50 mL), and the resulting yellow solid was filtered off to obtain pure compound **J**.

10.11.1. 2-Amino-1-(3-nitrophenyl)ethan-1-ol (J). Yield, 54%; yellow crystals; Rf (CH_2Cl_2:MeOH:Et_3N = 7:1:0.1): 0.29; ^{1}H NMR (DMSO-d_6): δ 4.72 (s, 2H, CH_2), 7.90 (dd, J = 8.1 Hz, 7.8 Hz, 1H, ArH), 8.45 (d, J = 7.8 Hz, 1H, ArH), 8.54 (d, J = 8.1 Hz, 1H, ArH), 8.61 (s, 3H, NH_3), 8.70 (s, 1H, ArH) ppm.

10.12. Synthesis of compound 12. Amine **J** (150 mg, 0.823 mmol, 1 equiv), indole-2-carboxylic acid (146 mg, 0.905 mmol, 1.1 equiv), HOBt (111 mg, 0.823 mmol, 1 equiv), and NMM (0.45 mL, 5 equiv) were dissolved in DMF (8 mL). EDC (189 mg, 0.988 mmol, 1.2 equiv) was added at 0 °C and the mixture was stirred at 0 °C for 24 h. The solvent was evaporated and ethyl acetate (100 mL) was added, followed by washing with 5% citric acid (2

× 25 mL), 1 M NaOH (2 × 25 mL), and brine (25 mL). The organic phase was dried over Na_2SO_4, filtered, and evaporated to dryness.

10.12.1. *N*-(2-Hydroxy-2-(3-nitrophenyl)ethyl)-1*H*-indole-2-carboxamide (12). Yield, 80%; yellow crystals; Rf (EtOAc): 0.60; m.p. 143-146 °C; ^{1}H NMR (DMSO-d_6): δ 3.47 (ddd, J = 13.1 Hz, 6.8 Hz, 5.3 Hz, 1H, CH_2), 3.55 (ddd, J = 13.1, 6.3, 5.6 Hz, 1H, CH_2), 4.94 (ddd, J = 6.8, 5.6, 4.6 Hz, 1H, CH), 5.94 (d, J = 4.6 Hz, 1H, OH), 7.02 (ddd, 8.2 Hz, 6.7 Hz, 1.0 Hz, 1H, Ar-H), 7.10 (d, J = 1.6 Hz, 1H, Ar-H), 7.16 (ddd, J = 8.2 Hz, 6.7 Hz, 1.0 Hz, 1H, Ar-H), 7.40 (dd, J = 8.2 Hz, 1.0 Hz, 1H, Ar-H), 7.60 (d, J = 8.2 Hz, 1H, Ar-H), 7.62 (dd, J = 8.0 Hz, 1H, Ar-H), 7.81 (d, J = 8.0 Hz, 1H, Ar-H), 8.12 (ddd, J = 8.2 Hz, 2.0, 0.8 Hz, 1H, Ar-H), 8.25 (dd, J = 2.0 Hz, 2.0 Hz, 1H, Ar-H), 8.58 (dd, J = 6.3 Hz, 5.3 Hz, 1H, NHCO), 11.54 (s, 1H,NH) ppm. ^{13}C NMR (DMSO-d_6): δ 46.7, 70.5, 102.7, 119.7, 120.7, 121.6, 122.1, 123.3, 127.1, 129.6, 131.6, 133.0, 136.5, 146.1, 147.7, 161.3 ppm. HRMS-ESI (*m/z*): calc for $C_{17}H_{14}N_3O_4$ 324.0984, found 324.0987. HPLC purity: 99% (t_r=6.169 min). IR (ATR): ν 3338, 3063, 2980, 2913, 2875, 2362, 1621, 1548, 1524, 1420, 1347, 1072, 741 cm^{-1}.

10.13. Synthesis of compound K. To a solution of 4-formylbenzoic acid (1.0 g, 6.66 mmol, 1 equiv) in THF, nitromethane (2.4 mL, 46.4 mmol, 7 equiv) was added. Then, LiOH (191 mg, 7.99 mmol, 1.2 equiv) and water (4 mL) were added, and the mixture was stirred for 3 days at room temperature. Ethyl acetate (50 mL) was added, and the mixture was washed with 1 M HCl (2 × 25 mL) and brine (25 mL). The organic phase was dried over Na_2SO_4, filtered, and evaporated to dryness.

10.13.1. 4-(1-Hydroxy-2-nitroethyl)benzoic acid (**K**). Yield, 51%; m.p. 160-163 °C; ^{1}H NMR (DMSO-d_6): δ 4.58 (dd, J = 12.6 Hz, 9.7 Hz, 1H, CH_2), 4.90 (dd, J = 12.6 Hz, 3.3 Hz, 1H, CH_2), 5.35 (ddd, J = 9.7 Hz, 5.0 Hz, 3.3 Hz, 1H, CH), 6.25 (d, J = 5.0 Hz, 1H, OH), 7.57 (d, J = 8.3 Hz, 2H, Ar-H), 7.93 (d, J = 8.3 Hz, 2H, ArH), 13.01 (bs, 1H, COOH) ppm. ^{13}C NMR (DMSO-d_6): δ 69.7, 81.6, 126.5, 129.5, 130.4, 145.4, 167.2 ppm. IR(ATR): ν 3497, 2908, 2668, 2550, 1681, 1609, 1570, 1420, 1380, 1284, 1215, 1081, 1014, 923, 863, 764, 716 cm^{-1}.

10.14. Synthesis of compound L. Compound **K** (600 mg, 1.84 mmol, 1 equiv) was dissolved in methanol (25 mL) and purged with argon. Pd/C (60 mg) and di-*tert*-butyl dicarbonate (Boc_2O) (682 mg, 3.12 mmol, 1.7 equiv) were added, and the mixture was stirred under a hydrogen atmosphere for 3.5 h. After filtration to remove Pd/C, the solvent was evaporated, and ethyl acetate was added to the residue. The organic phase was washed with 1 M NaOH (2 × 30 mL) and then with water until neutral. The aqueous phase was then acidified to pH 2 with an appropriate acid (the acid used for acidification should be specified, typically HCl), and extracted with ethyl acetate (2 × 30 mL). The combined ethyl acetate extracts were washed with brine (25 mL) and dried over Na_2SO_4. After filtration, the solvent was evaporated. The crude product was recrystallized from a mixture of ethyl acetate, diethyl ether, and petroleum ether, and further purified by column chromatography using CH_2Cl_2 (dichloromethane): MeOH (methanol): CH_3COOH (acetic acid) = 20:1:0.1 as mobile phase.

10.14.1. 4-(2-((*tert*-Butoxycarbonyl)amino)-1-hydroxyethyl)benzoic acid (L). White crystals; Yield, 49%; m.p. 68-70 °C; ^{1}H NMR (DMSO-d_6): δ 1.22 (s, 9H, CH_3), 3.52 (dd, J = 6.8 Hz, 1.2 Hz, 2H, CH_2), 4.84 (td, J = 6.8 Hz, 4.6 Hz, 1H, CH), 5.55 (d, J = 4.6 Hz, 1H, OH), 7.45 (d, J = 8.2 Hz, 2H, ArH), 7.89 (d, J = 8.2 Hz, 2H, ArH), 9.32 (s, 1H, NH), 12.65 (bs, 1H,

COOH). ^{13}C NMR (DMSO-d_6): δ 28.5, 58.8, 71.5, 82.2, 127.6, 130.9, 131.6, 149.4, 158.1, 170.1 ppm. IR (ATR): ν 3239, 2978, 2931, 1683, 1402, 1239, 1163, 1107, 852, 765, 701 cm^{-1}.

10.15. Synthesis of compound M. To a solution of compound L (300 mg, 1.07 mmol, 1 equiv) in methanol (40 mL), thionyl chloride ($SOCl_2$) (85 μL, 1.17 mmol, 1.1 equiv) was added and the mixture was stirred for 12 hours. After the reaction, the solvent was evaporated under reduced pressure. The residue was crystallized from a mixture of methanol, diethyl ether and ethyl acetate.

10.15.1. Methyl 4-(2-amino-1-hydroxyethyl)benzoate hydrochloride (**M**). Yellow crystals; Yield, 63%; Rf(CH_2Cl_2: MeOH: CH_3COOH =20:1:0.1) = 0.41; m.p. 217-219 °C; ^1H NMR (DMSO-d_6): δ 3.23 (dd, J = 12.6 Hz, 10.2 Hz, 1H, CH_2), 3.34 (dd, J = 12.6 Hz, 2.8 Hz, 1H, CH_2), 3.86 (s, 3H, CH_3), 5.11 (dd, J = 10.2 Hz, 2.8 Hz, 1H, CH), 6.31 (bs, 1H, OH), 7.56 (d, J = 8.4 Hz, 2H, ArH), 7.98 (d, J = 8.4 Hz, 2H, ArH) ppm. IR(ATR): 3416, 2944, 1688, 1605, 1486, 1419, 1380, 1282, 1225, 1110, 1005, 852, 767, 701 cm^{-1}. HRMS for $C_{10}H_{14}NO_3$: calculated 196.0974; found 196.0969.

10.16. Synthesis of compound 17. Compound **17** was synthesized by the same coupling procedure as compound **12**, starting from compound **M** and 4,5-dibromo-1*H*-pyrrolo[2,3-*b*]pyridine-2-carboxylic acid. The crude product was purified by column chromatography on silica gel using a solvent system of dichloromethane (CH_2Cl_2) and methanol (MeOH) in a ratio of 20:1.

10.16.1. Methyl 4-(2-(4,5-dibromo-1*H*-pyrrole-2-carboxamido)-1-hydroxyethyl)benzoate (**17**). White crystals; Yield, 53%; Rf(CH_2Cl_2: MeOH=20:1) = 0.20; m.p. 198-200 °C; ^1H NMR (DMSO-d_6) δ 3.73 (dd, J = 13.7 Hz, 5.8 Hz, 1H, CH_2), 3.84 (s, 3H, CH_3), 3.87 (dd, J = 13.7

Hz, 7.6 Hz, 1H, CH_2), 4.99 (ddd, J = 7.6 Hz, 5.8 Hz, 4.7 Hz, 1H, CH), 5.69 (d, J = 4.7 Hz, 1H, OH), 6.91 (s, 1H, ArH), 7.51 (d, J = 8.3 Hz, 2H, ArH), 7.91 (d, J = 4.7 Hz, 1H, OH), 6.91 (s, 1H, ArH) ppm. ^{13}C NMR (DMSO-d_6) δ 52.1, 55.9, 68.8, 98.1, 105.5, 117.1, 125.9, 126.4, 128.4, 129.0, 148.9, 159.0, 166.1 ppm. IR(ATR): ν 3402, 3161, 2997, 1880, 1715, 1591, 1542, 1463, 1407, 1279, 1110, 975, 770, 703 cm^{-1}. HRMS for $C_{15}H_{15}Br_2N_2O_4$: calculated 444.9393; found 444.9395. HPLC: t_r = 5.990 min (99.0% at 254 nm).

10.17. Synthesis of compound O. Methyl *L*-phenylalaninate (0.95 g, 2.6 mmol) and 2,2,2-trichloro-1-(4,5-dibromo-1*H*-pyrrol-2-yl)ethan-1-one (0.60 g, 2.8 mmol) were dissolved in 15 mL of acetonitrile and stirred for 3 days at room temperature under an argon atmosphere. The organic solvent was evaporated under reduced pressure and ethyl acetate (50 mL) was added to the residue. This mixture was washed with hydrochloric acid (2 × 25 mL) and saturated solution of sodium chloride (50 mL). The organic layer was dried over Na_2SO_4, filtered, and evaporated to dryness. The crude residue was purified by column chromatography using hexane and ethyl acetate in a 7:3 ratio with 2% acetic acid as mobile phase. The final purification was achieved using chloroform ($CHCl_3$) to obtain white crystals.

10.17.1. Methyl (4,5-dibromo-1*H*-pyrrole-2-carbonyl)-*L*-phenylalaninate (O). Yield, 70%; white crystals; m.p. 134-136 °C; ^{1}H NMR (DMSO-d_6): δ 3.00 (dd, J= 13.8 Hz, 10.2 Hz, 1H, CH_2), 3.13 (dd, J = 13.8 Hz, 5.1 Hz, 1H, CH_2), 3.63 (s, 3H, CH_3), 4.16 (ddd, J= 10.2 Hz, 8.0 Hz, 5.1 Hz, 1H, CH), 7.06 (d, J = 2.8 Hz, 1H, ArH), 7.16-7.22 (m, 1H, ArH), 7.24-7.30 (m, 4H, Ar-H), 8.51 (d, J= 8.0 Hz, 1H, NH), 12.69 (s, 1H, NH), 12.69 (bs, 1H, NH) ppm. ^{13}C NMR (DMSO-d_6): δ 36.4, 52.0, 63.7, 97.9, 105.2, 113.3, 126.5, 127.3, 128.3, 129.1, 137.5, 158.8, 172.1 ppm. IR (ATR): ν 3352, 3117, 2952, 2860, 1718, 1630, 1557, 1505, 1436, 1400, 1356,

1217, 1101, 974, 745, 698 cm^{-1}. HRMS for $C_{15}H_{13}N_2O_3Br_2$:calculated 426.9293; found 426.9298. HPLC: t_r = 9.401 min (99.9% at 254 nm). $[\alpha]_D$ = -55.7 (c 1.00, MeOH).

10.18. Synthesis of compound 14. To a solution of compound **O** (1.28 g, 2.9 mmol) in 1,4-dioxane (25 mL), 1 M LiOH solution (25 mL) was added and the mixture was stirred overnight at room temperature. Water (100 mL) was added and the aqueous phase was extracted with ethyl acetate (100 mL). The aqueous layer was then acidified with 5 M HCl to pH 1 and extracted with ethyl acetate (3×50 mL). The combined organic layers were dried over anhydrous Na_2SO_4, filtered, and the solvent was removed under reduced pressure. The crude product was crystallized from chloroform ($CHCl_3$) to yield compound **14** as the final product.

(4,5-Dibromo-1*H*-pyrrole-2-carbonyl)-*L*-phenylalanine (**14**). Brown crystals; m.p. 200-203 ˚C; ^{1}H NMR (DMSO-d_6): δ 2.84 (dd, J = 13.8 Hz, 10.4 Hz, 1H, CH_2), 3.02 (dd, J = 13.8 Hz, 4.4 Hz, 1H, CH_2), 4.46 (ddd, J = 10.4 Hz, 8.4 Hz, 1H, CH), 6.63 (d, J = 8.5 Hz, 2H, ArH), 7.01 (d, J = 2.6 Hz, 1H, NH), 7.05 (d, J = 8.5 Hz, 2H, ArH), 8.29 (d, J = 8.4 Hz, 1H, CONH), 9.19 (s, 1H, OH), 12.66 (d, J = 2.6 Hz, 1H, NH), 12.71 (bs, 1H, COOH) ppm; ^{13}C NMR (DMSO-d_6): δ 35.8, 54.0, 97.9, 104.9, 113.2, 115.0, 127.6, 128.0, 130.0, 155.9, 158.8, 173.3 ppm. IR(ATR): ν 3266, 1720, 1626, 1554, 1511, 1416, 1210, 973, 829, 758, 646 cm^{-1}. HRMS for $C_{14}H_{11}N_2O_4Br_2$: calculated 428.9098; found 428.9094. $[\alpha]_D$=+2.9 (c 1.00, MeOH).

11. ^{1}H NMR, ^{13}C NMR spectra and HPLC chromatograms of the representative tested compounds

11.1 Figure S6.

^{1}H NMR (400 MHz, DMSO-d_6, 25°C, TMS)

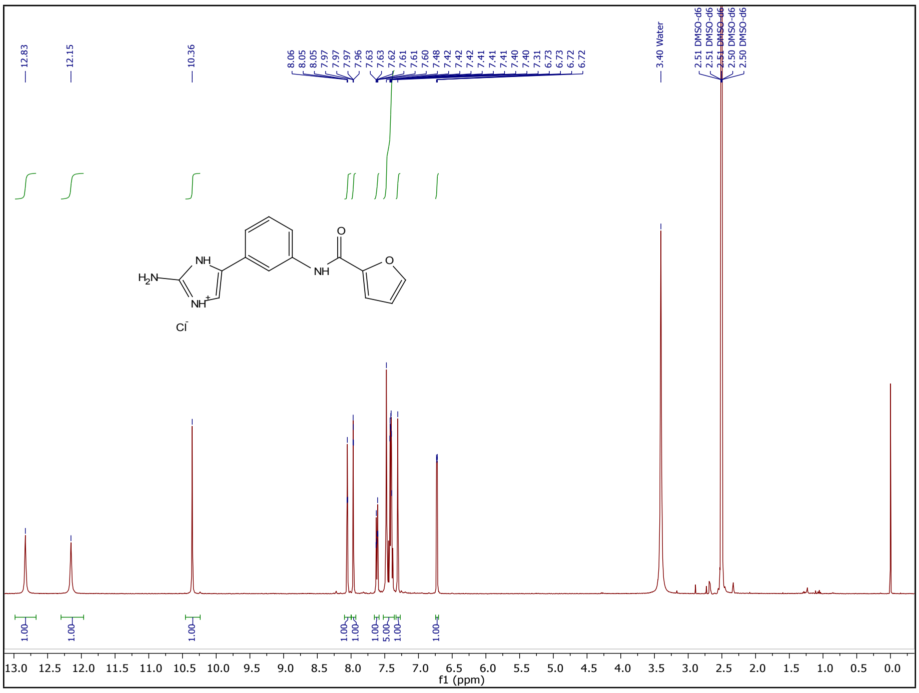

^{1}H NMR spectrum of compound **30**.

11.2 Figure S7.

^{13}C NMR (101 MHz, DMSO-d_6, 25 ˚C, TMS)

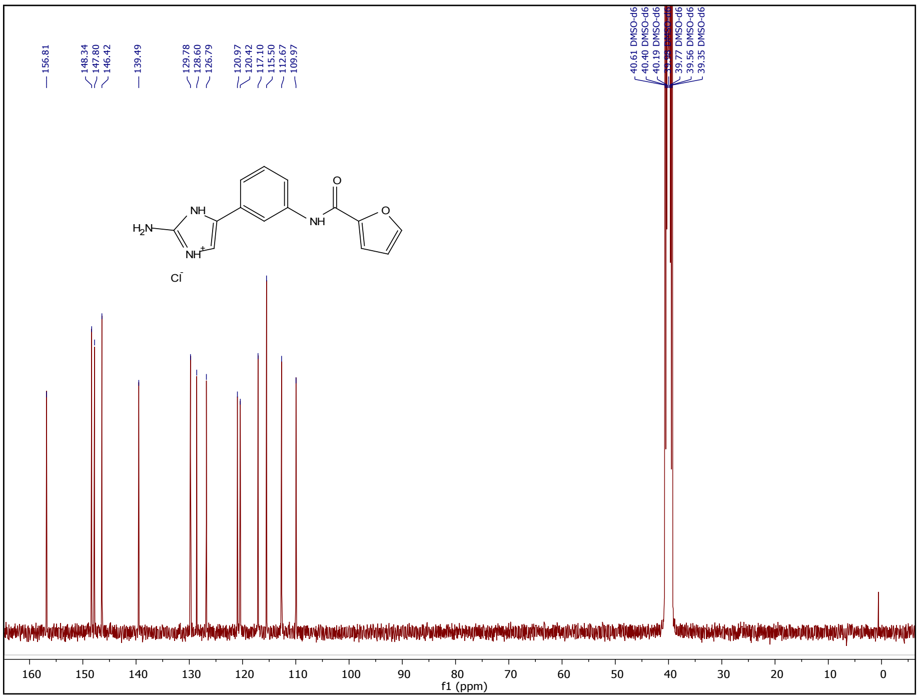

^{13}C NMR spectrum of compound **30**.

11.3 Figure S8.

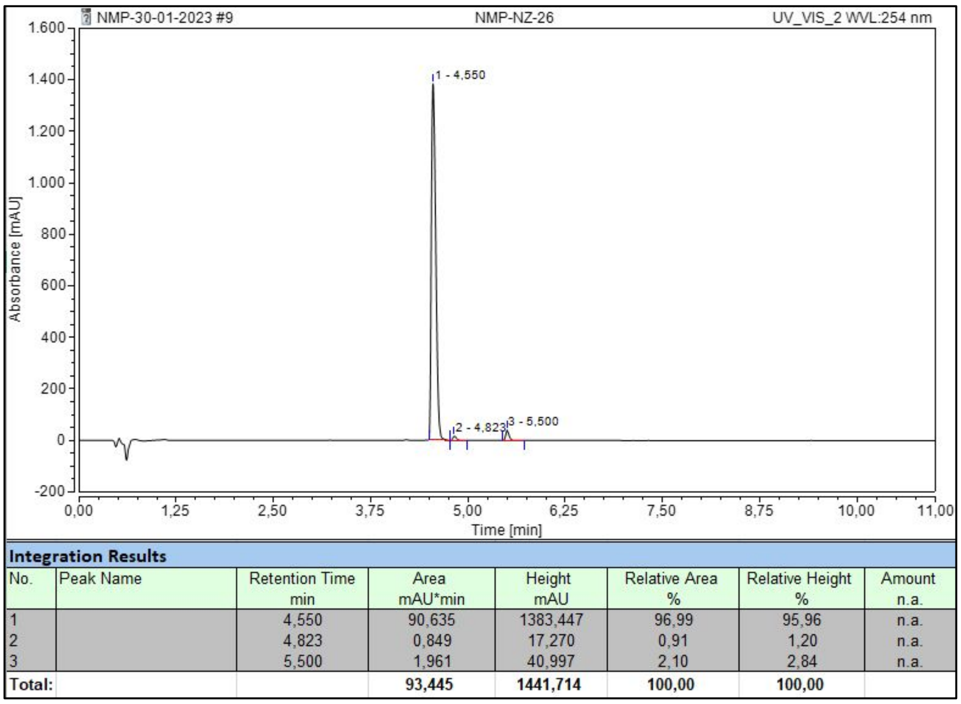

Integration Results

No.	Peak Name	Retention Time min	Area mAU*min	Height mAU	Relative Area %	Relative Height %	Amount n.a.
1		4,550	90,635	1383,447	96,99	95,96	n.a.
2		4,823	0,849	17,270	0,91	1,20	n.a.
3		5,500	1,961	40,997	2,10	2,84	n.a.
**Total:**			**93,445**	**1441,714**	**100,00**	**100,00**	

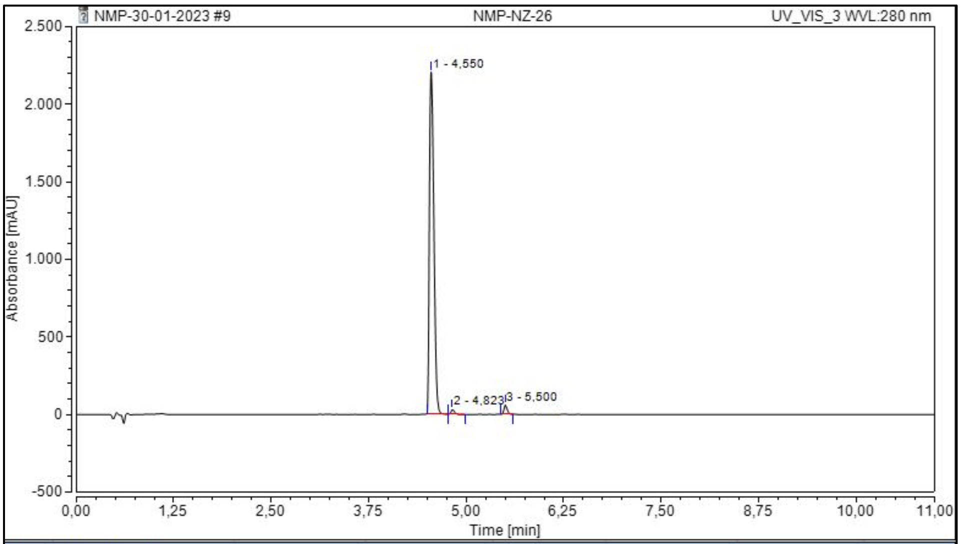

Integration Results

No.	Peak Name	Retention Time min	Area mAU*min	Height mAU	Relative Area %	Relative Height %	Amount n.a.
1		4,550	146,083	2205,585	97,07	95,97	n.a.
2		4,823	1,526	31,157	1,01	1,36	n.a.
3		5,500	2,878	61,479	1,91	2,68	n.a.
**Total:**			**150,487**	**2298,221**	**100,00**	**100,00**	

HPLC chromatograms for compound **30**.

11.4 Figure S9.

^{1}H NMR (400 MHz, DMSO-d_6, 25˚C, TMS)

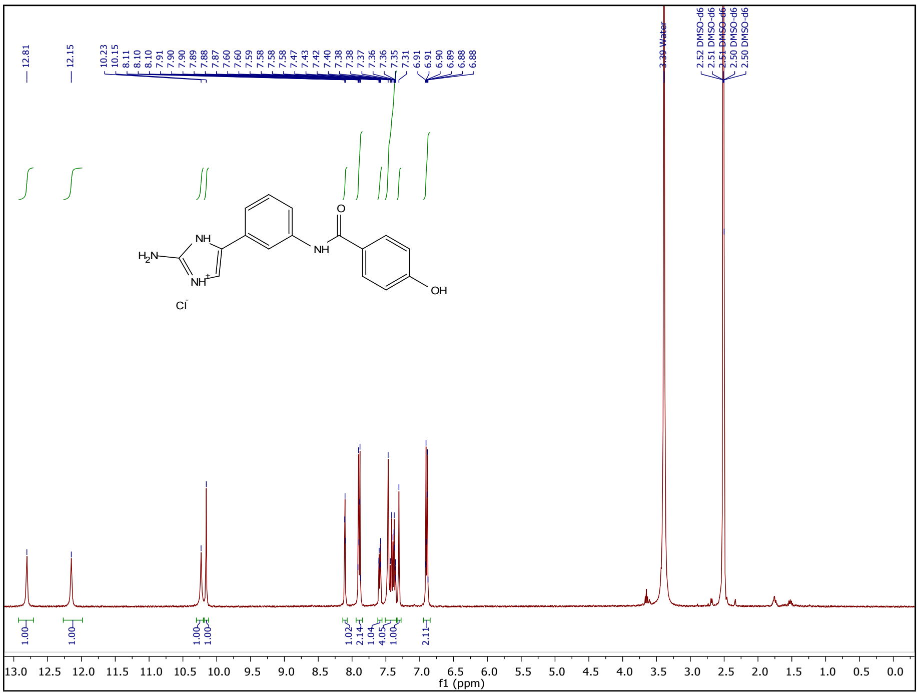

^{1}H NMR spectrum of compound **37**.

11.5 Figure S10.

^{13}C NMR (101 MHz, DMSO-d_6, 25 ˚C, TMS)

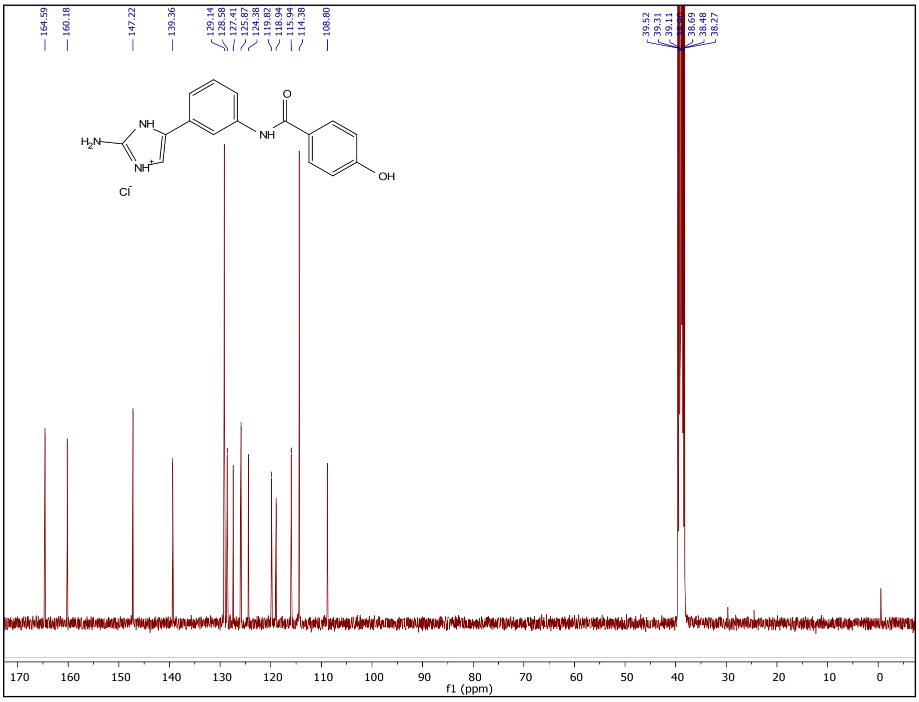

^{13}C NMR spectrum of compound **37**.

11.6 Figure S11.

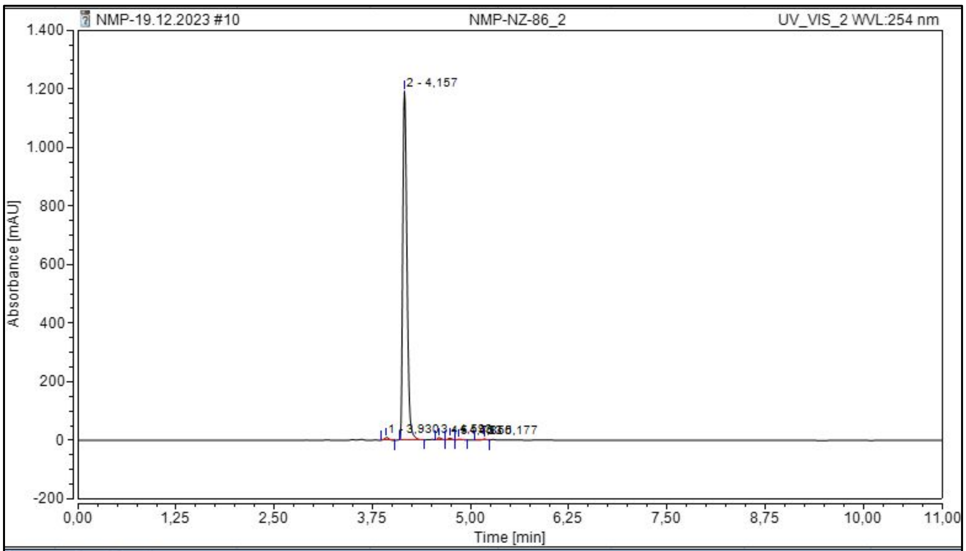

Integration Results

No.	Peak Name	Retention Time min	Area mAU*min	Height mAU	Relative Area %	Relative Height %	Amount n.a.
1		3,930	0,596	9,647	0,79	0,79	n.a.
2		4,157	73,872	1191,500	97,77	97,61	n.a.
3		4,593	0,423	8,015	0,56	0,66	n.a.
4		4,733	0,316	6,108	0,42	0,50	n.a.
5		4,850	0,161	2,319	0,21	0,19	n.a.
6		5,177	0,190	3,140	0,25	0,26	n.a.
**Total:**			**75,557**	**1220,729**	**100,00**	**100,00**	

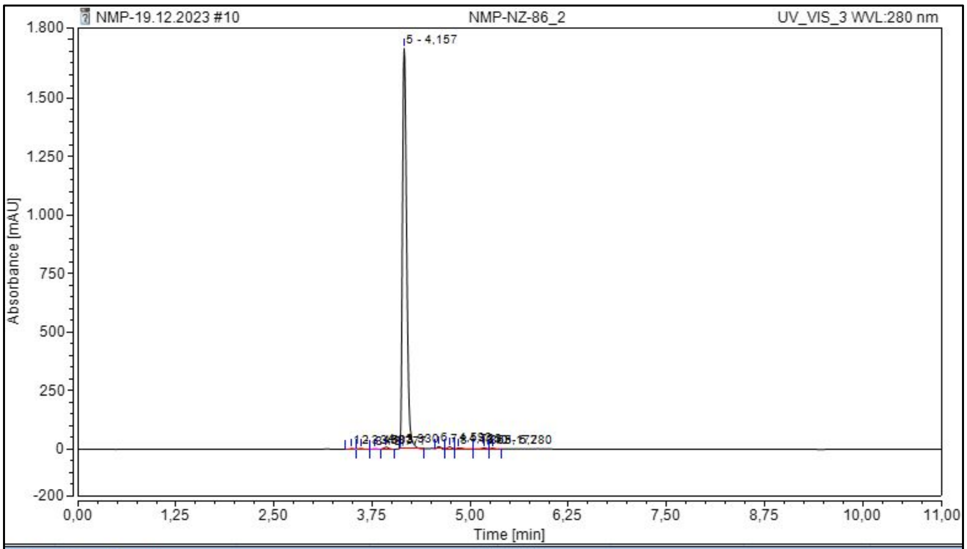

Integration Results

No.	Peak Name	Retention Time min	Area mAU*min	Height mAU	Relative Area %	Relative Height %	Amount n.a.
1		3,490	0,164	2,823	0,15	0,16	n.a.
2		3,603	0,207	2,796	0,19	0,16	n.a.
3		3,777	0,051	0,971	0,05	0,06	n.a.
4		3,930	0,631	9,260	0,58	0,53	n.a.
5		4,157	106,266	1710,671	97,40	97,35	n.a.
6		4,593	0,535	10,191	0,49	0,58	n.a.
7		4,733	0,480	9,040	0,44	0,51	n.a.
8		4,853	0,272	3,665	0,25	0,21	n.a.
9		5,177	0,295	4,365	0,27	0,25	n.a.
10		5,280	0,199	3,539	0,18	0,20	n.a.
**Total:**			**109,099**	**1757,319**	**100,00**	**100,00**	

HPLC chromatograms for compound **37**.

11.7 Figure S12.

^{1}H NMR (400 MHz, DMSO-d_6, 25°C, TMS)

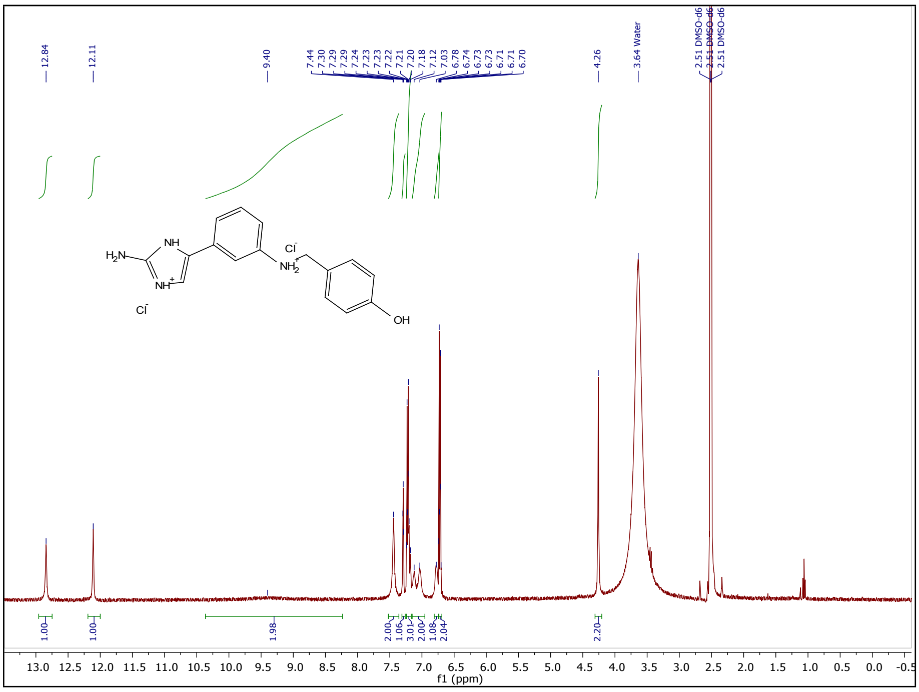

^{1}H NMR spectrum of compound **43**.

11.8 Figure S13.

^{13}C NMR (101 MHz, DMSO-d_6, 25 ˚C, TMS)

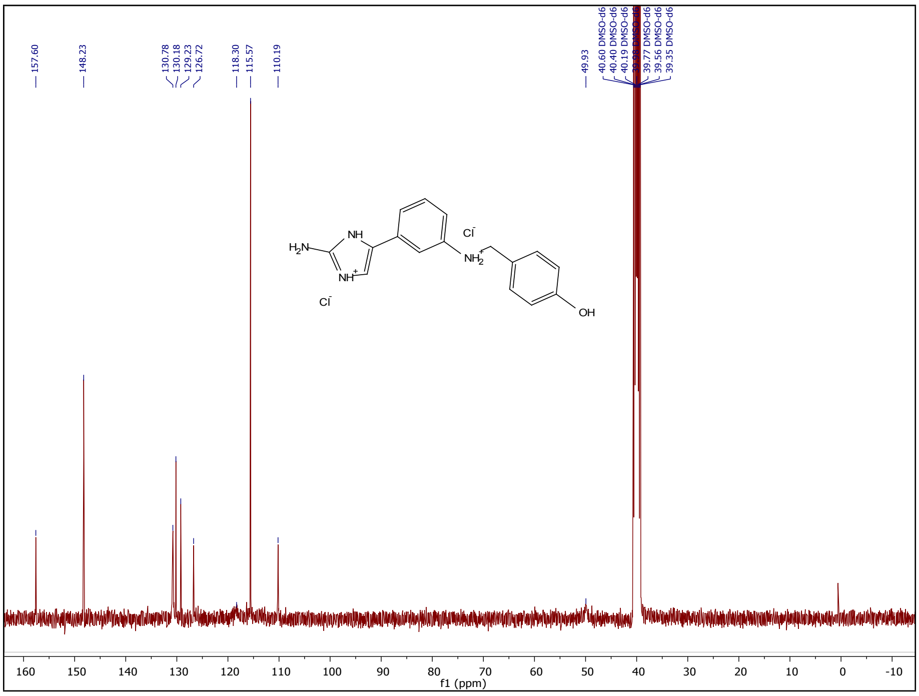

^{13}C NMR spectrum of compound **43**.

11.9 Figure S14.

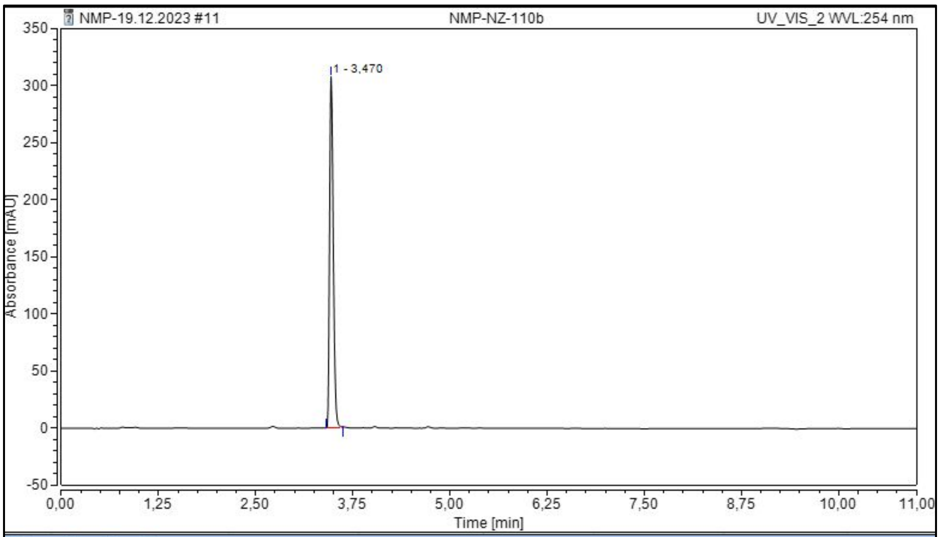

Integration Results

No.	Peak Name	Retention Time<br>min	Area<br>mAU*min	Height<br>mAU	Relative Area<br>%	Relative Height<br>%	Amount<br>n.a.
1		3,470	16,872	307,536	100,00	100,00	n.a.
**Total:**			**16,872**	**307,536**	**100,00**	**100,00**	

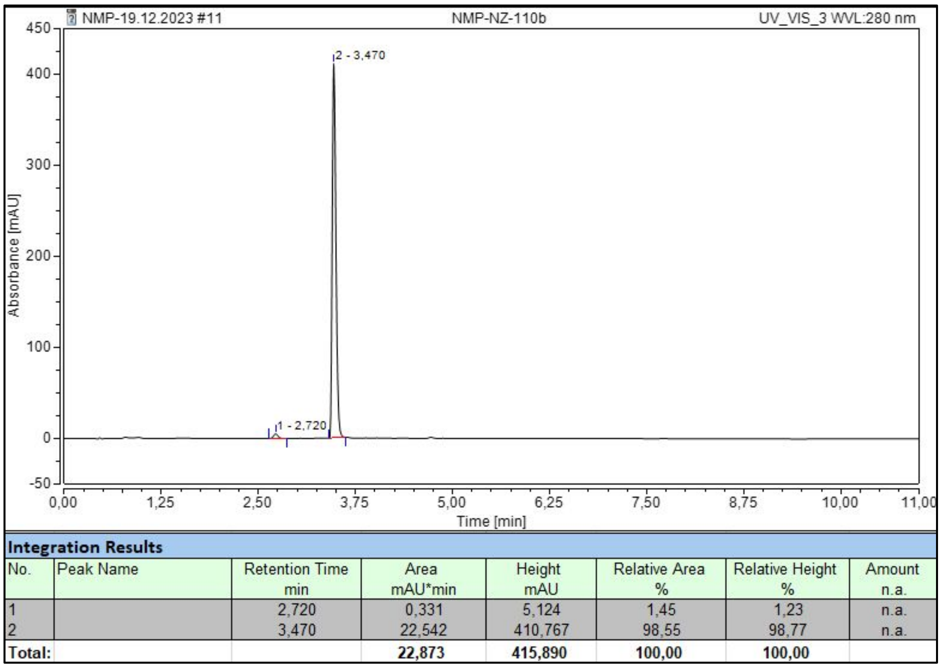

Integration Results

No.	Peak Name	Retention Time min	Area mAU*min	Height mAU	Relative Area %	Relative Height %	Amount n.a.
1		2,720	0,331	5,124	1,45	1,23	n.a.
2		3,470	22,542	410,767	98,55	98,77	n.a.
**Total:**			**22,873**	**415,890**	**100,00**	**100,00**	

HPLC chromatograms for compound **43**.

11.10 Figure S15.

^{1}H NMR (400 MHz, DMSO-d_6, 25°C, TMS)

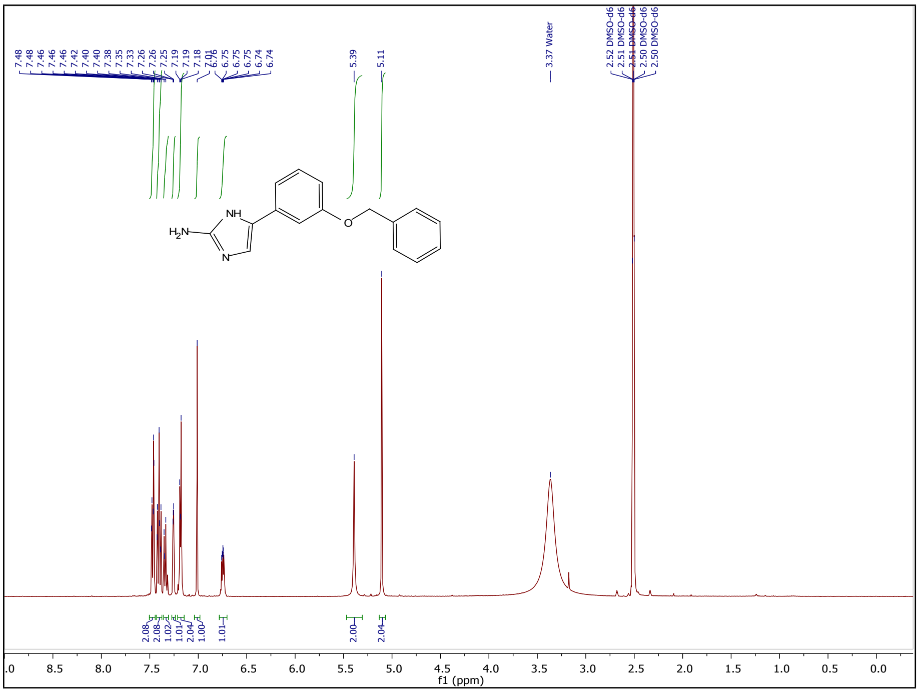

^{1}H NMR spectrum of compound **42**.

11.11 Figure S16.

^{13}C NMR (101 MHz, DMSO-d_6, 25 °C, TMS)

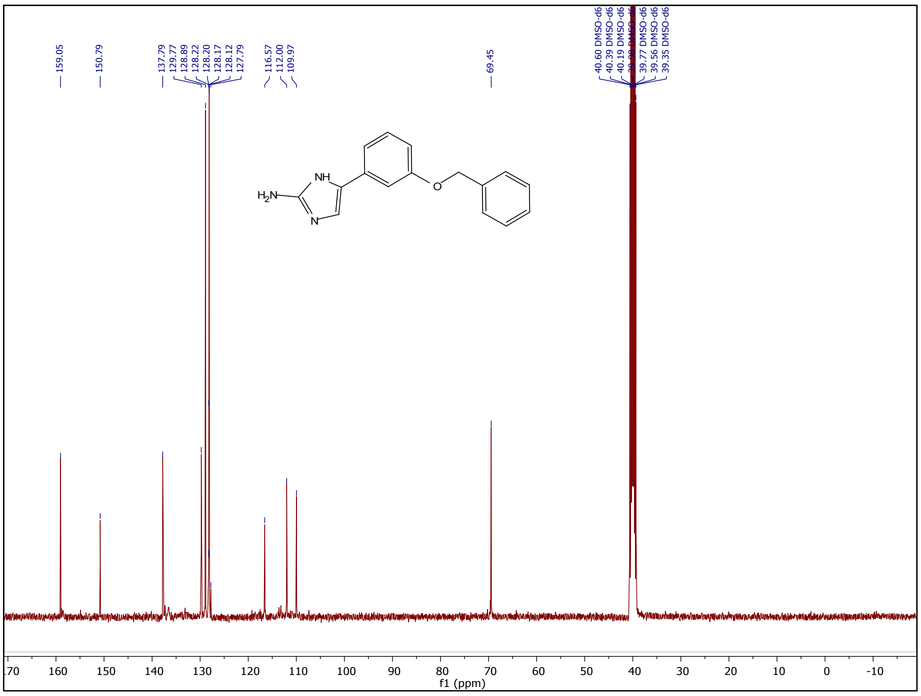

^{13}C NMR spectrum of compound **42**.

11.12 Figure S17.

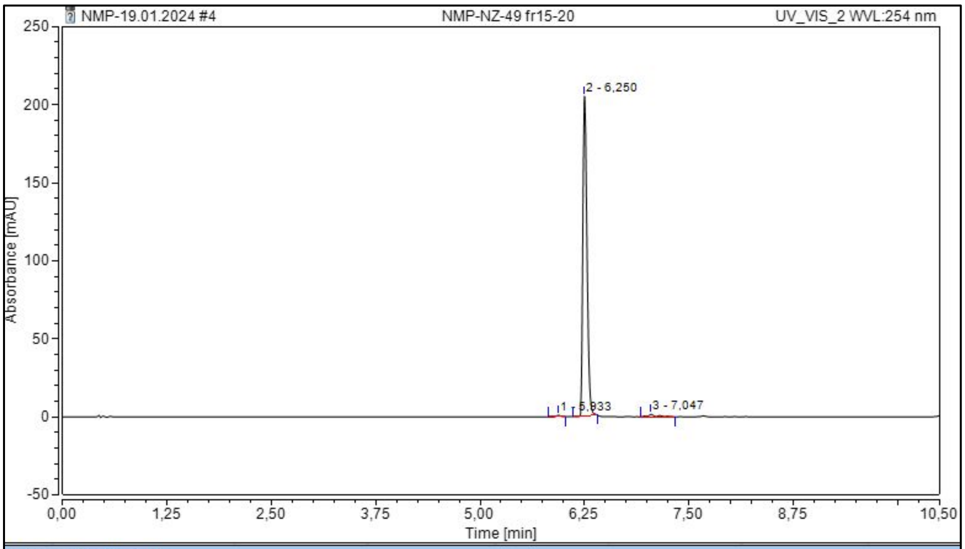

Integration Results

No.	Peak Name	Retention Time min	Area mAU*min	Height mAU	Relative Area %	Relative Height %	Amount n.a.
1		5,933	0,051	1,020	0,42	0,49	n.a.
2		6,250	11,844	204,971	98,15	98,70	n.a.
3		7,047	0,172	1,683	1,43	0,81	n.a.
**Total:**			**12,067**	**207,674**	**100,00**	**100,00**	

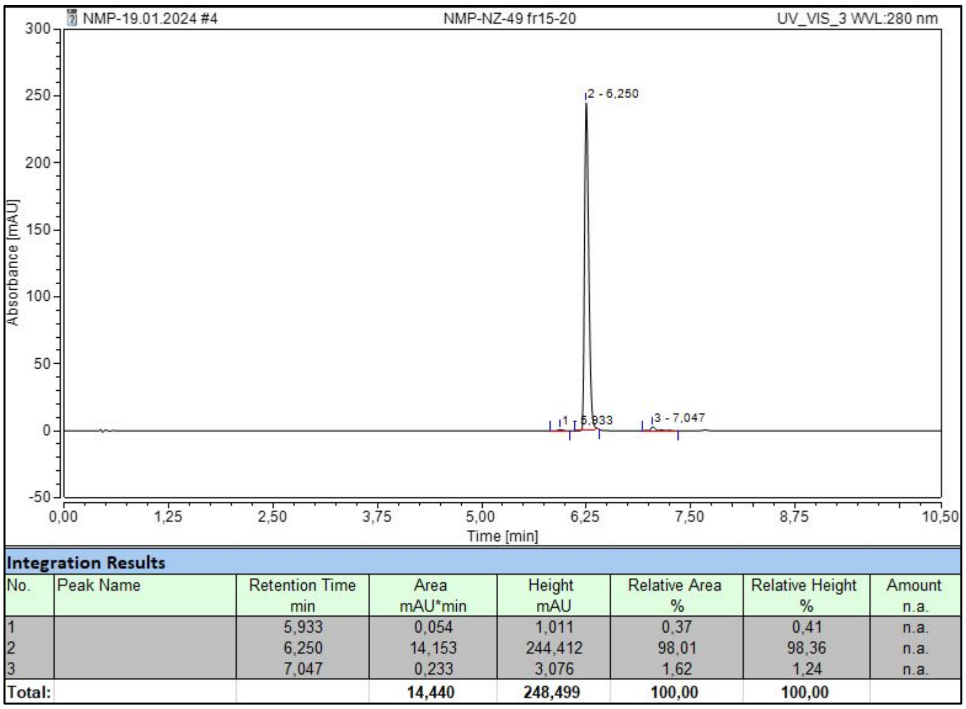

Integration Results

No.	Peak Name	Retention Time min	Area mAU*min	Height mAU	Relative Area %	Relative Height %	Amount n.a.
1		5,933	0,054	1,011	0,37	0,41	n.a.
2		6,250	14,153	244,412	98,01	98,36	n.a.
3		7,047	0,233	3,076	1,62	1,24	n.a.
**Total:**			**14,440**	**248,499**	**100,00**	**100,00**	

HPLC chromatograms for compound **42**.

11.13 Figure S18.

^{1}H NMR (400 MHz, DMSO-d_6, 25˚C, TMS)

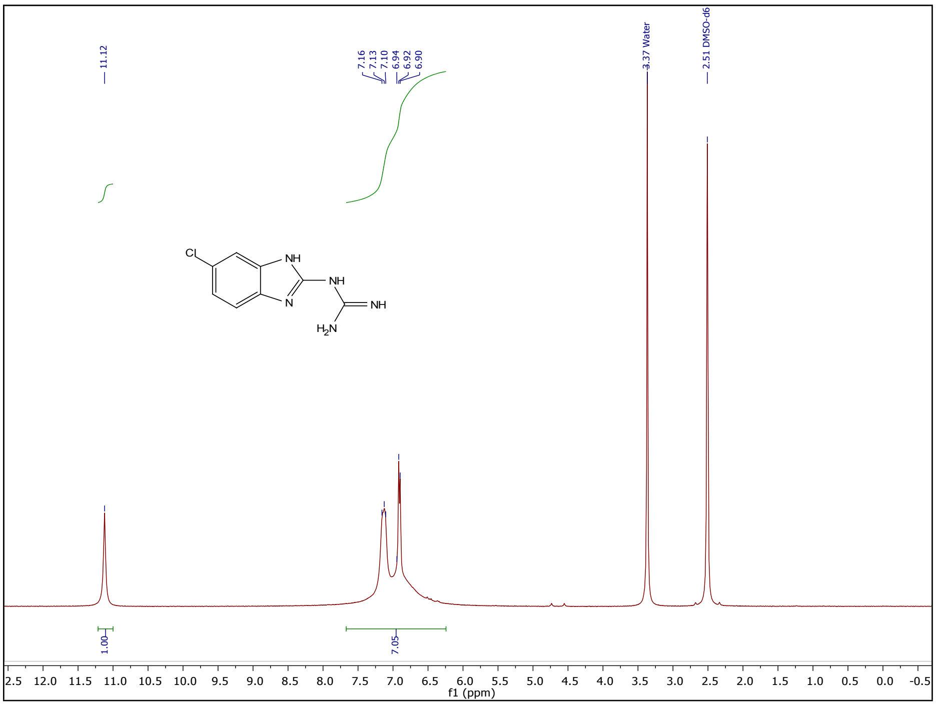

^{1}H NMR spectrum of compound **ClGBI**.

11.14 Figure S19.

^{13}C NMR (101 MHz, DMSO-d_6, 25 ˚C, TMS)

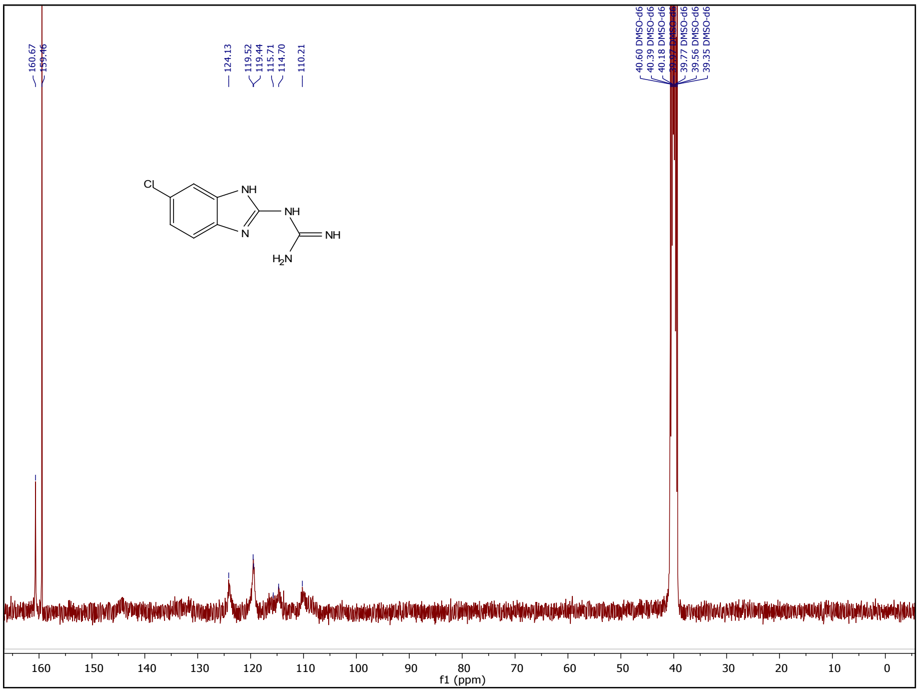

^{13}C NMR spectrum of compound **ClGBI**.

11.15 Figure S20.

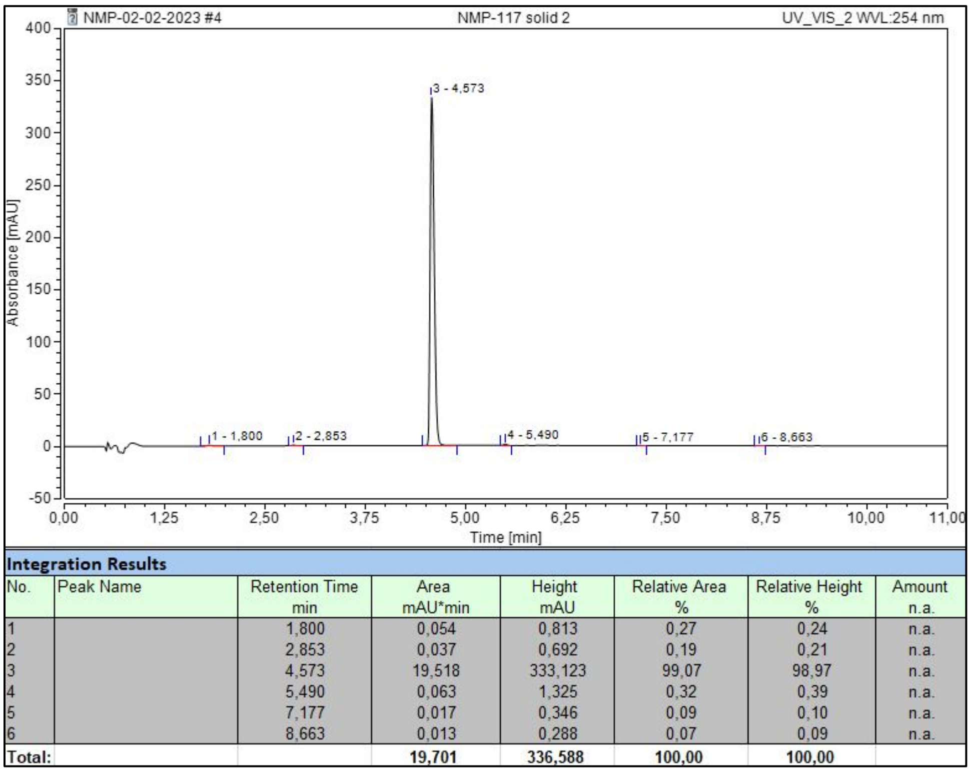

Integration Results

No.	Peak Name	Retention Time min	Area mAU*min	Height mAU	Relative Area %	Relative Height %	Amount n.a.
1		1,800	0,054	0,813	0,27	0,24	n.a.
2		2,853	0,037	0,692	0,19	0,21	n.a.
3		4,573	19,518	333,123	99,07	98,97	n.a.
4		5,490	0,063	1,325	0,32	0,39	n.a.
5		7,177	0,017	0,346	0,09	0,10	n.a.
6		8,663	0,013	0,288	0,07	0,09	n.a.
**Total:**			**19,701**	**336,588**	**100,00**	**100,00**	

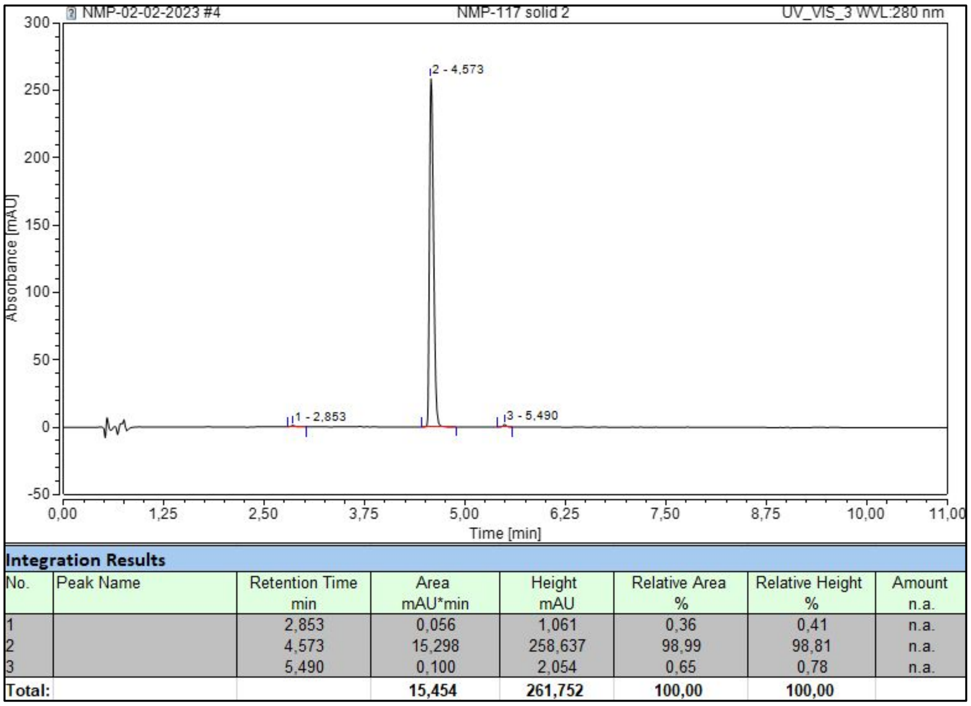

Integration Results

No.	Peak Name	Retention Time min	Area mAU*min	Height mAU	Relative Area %	Relative Height %	Amount n.a.
1		2,853	0,056	1,061	0,36	0,41	n.a.
2		4,573	15,298	258,637	98,99	98,81	n.a.
3		5,490	0,100	2,054	0,65	0,78	n.a.
**Total:**			**15,454**	**261,752**	**100,00**	**100,00**	

HPLC chromatograms for **ClGBI**.

12. SMILES for all tested compounds

Comp.	SMILES
**1**	FC(F)(F)C1=CC=CC=C1N2CCC(NC(CC3=CC=NN3)=O)C2
**2**	CN(CCC1CCN(S(=O)(C2=CNC=N2)=O)CC1)C
**3**	CC1=CN=C(CN2CCCC(C3=NNC(C(N)=O)=C3)C2)C=N1
**4**	N1(CC2=NNC=C2)CCCC1C3=NC(CCCC4)=C4N3
**5**	CC(N1CCCC(N2CCCCCC2)C1)C(O)=O
**6**	CC1=C(C)OC(CN2CCCC(C3=CC=NN3)C2)=N1
**7**	FC1=C(C=CC=C1OC)CN2C[C@@H]3CNC[C@@H]3C2
**8**	CC(N1CCC(C2=CC3=C(N2)C=CC=C3)CC1)C(N)=O
**9**	CC(O)C1CCCN(CC2=CC=CC3=C2C=CN3)C1
**10**	NC(CC1)CCN1CC2=CNC3=C2C=CC=C3
**11**	NC1=NC(/C=C/CNC(C2CC(C=CC=C3)=C3N2)=O)=CN1
**12**	O=C(NCC(O)C1=CC=CC([N+]([O-])=O)=C1)C2=CC3=C(C=CC=C3)N2
**13**	NC1=NC=C(C2=CC=CC(NC(C3=CC4=C(N3)C=CC(OC(F)(F)F)=C4)=O)=C2)N1
**14**	O=C(N[C@H](C(O)=O)CC1=CC=CC=C1)C2=CC(Br)=C(Br)N2
**15**	O=C(C1=CC(Br)=C(Br)N1)NC2=CC=C(N3C[C@@H](C(O)=O)CCC3)C=C2
**16**	O=C(C1=CC2=C(C=CC=C2)N1)NC3=CC=C(OCC(OC)=O)C=C3
**17**	OC(CNC(C1=CC(Br)=C(Br)N1)=O)C2=CC=C(C(OC)=O)C=C2
**18**	CN1C2=CC=C(NC(C3=CC(Br)=C(Br)N3)=O)C=C2OC(C)(CNC(C(O)=O)=O)C1
**19**	NC1=NC(CCC(CNC(C2=CC(Br)=CN2)=O)C3)=C3S1
**20**	O=C(NC[C@H]1CC[C@@H](C(NCC(O)=O)=O)CC1)C2=CC(Br)=C(Br)N2
**21**	O=C(NC1=CC=CC(C2=CN=C(N)N2C(OC(C)(C)C)=O)=C1)C3=CC(C=C(OC)C=C4)=C4N3
**22**	O=C(NC1=CC=CC(C2=CN=C(N)N2C(OC(C)(C)C)=O)=C1)C3=CC(C=C(F)C=C4)=C4N3
**23**	NC1=NC=C(C2=CC=CC(NC(C3=CC4=C(N3)C=CC(OC)=C4)=O)=C2)N1
**24**	NC1=NC=C(C2=CC=CC(NC(C3=CC4=C(N3)C=CC(F)=C4)=O)=C2)N1
**25**	NC1=NC=C(C2=CC=CC(NC(C3=CC4=C(N3)C=CC=C4)=O)=C2)N1
**26**	NC1=NC=C(C2=CC=CC(NC(C3=CC=CN3)=O)=C2)N1
**27**	NC1=NC=C(C2=CC=CC(NC(C3=CC=CN3)=O)=C2)N1
**28**	NC1=[NH+]C=C(N1)C2=CC=CC(NC(C3=CNC4=C3C=CC=C4)=O)=C2.[Cl-]
**29**	O=C(C1=CNC=C1)NC2=CC=CC(C3=CN=C(N3)N)=C2
**30**	NC1=NC=C(C2=CC=CC(NC(C3=CC=CO3)=O)=C2)N1
**31**	O=C(NC1=CC(C2=CN=C(N2)NC)=CC=C1)C3=CC4=C(N3)C=CC=C4
**32**	O=C(NC1=CC(C2=CN=C(N2)N)=CC=C1)C3=CC4=C(N3)C=CC(OCC5=CC=CC=C5)=C4
**33**	NC1=NC=C(C2=CC=CC(NC(C3=CC4=C(N3)C=CC(O)=C4)=O)=C2)N1
**34**	NC1=NC=C(C2=CC=CC(NC(C3=CC4=C(N3)C=CC(Cl)=C4)=O)=C2)N1
**35**	O=C(NC1=CC(C2=CN=C(N2)N)=CC=C1)C3=CC4=C(N3)C=CS4
**36**	NC1=NC=C(C2=CC=CC(NC(C3=CC4=C(N3)C=CC(N)=C4)=O)=C2)N1
**37**	NC1=NC=C(C2=CC=CC(NC(C3=CC=C(C=C3)O)=O)=C2)N1
**38**	NC1=NCC(C2=CC=CC(NC(C3=CC4=C(N3)C=CC=C4)=O)=C2)N1
**39**	O=C(NC1=CC(C2NC(NC)=NC2)=CC=C1)C3=CC=CO3
**40**	O=C(NC1=CC(C2NC(NC)=NC2)=CC=C1)C3=CC=CN3
**41**	O=C(C1=CC(C=CC=C2)=C2N1)NC3=CC(C4N(C(NC)=NC4)C(OC(C)(C)C)=O)=CC=C3
**42**	NC1=NC=C(N1)C2=CC=CC(OCC3=CC=CC=C3)=C2
**43**	NC1=NC=C(C2=CC=CC(NCC3=CC=C(C=C3)O)=C2)N1
**44**	NC1=NC(CCCNC(C2=CC(C=CC=C3)=C3N2)=O)=CN1

13. References

(1) Geragotelis, A. D.; Wood, M. L.; Göddeke, H.; Hong, L.; Webster, P. D.; Wong, E. K.; Freites, J. A.; Tombola, F.; Tobias, D. J. Voltage-dependent structural models of the human Hv1 proton channel from long-timescale molecular dynamics simulations. *Proc Natl Acad Sci U S A* **2020**, *117* (24), 13490-13498. DOI: 10.1073/pnas.1920943117 From NLM.

(2) Blau, L.; Menegon, R. F.; Trossini, G. H. G.; Molino, J. V. D.; Vital, D. G.; Cicarelli, R. M. B.; Passerini, G. D.; Bosquesi, P. L.; Chin, C. M. Design, synthesis and biological evaluation of new aryl thiosemicarbazone as antichagasic candidates. *Eur J Med Chem* **2013**, *67*, 142-151. DOI: 10.1016/j.ejmech.2013.04.022.

(3) Xie, W. L.; Sun, S. M.; Xu, J. X. Experimental Evidence on the Formation of Highly Strained 6,7-Dihydroazeto[2,1-b]oxazol-3-ium Derivatives as Reactive Intermediates. *Helv Chim Acta* **2022**, *105* (2). DOI: ARTN e202100187. 10.1002/hlca.202100187.
